# Selective Targeting of a Defined Subpopulation of Corticospinal Neurons Using a Novel Klhl14-Cre Mouse Line Enables Molecular and Anatomical Investigations through Development into Maturity

**DOI:** 10.1523/ENEURO.0589-24.2025

**Published:** 2025-09-03

**Authors:** Jake Lustig, Alexander Lammers, Julia Kaiser, Payal Patel, Aidan Raghu, James M. Conner, Phong Nguyen, Eiman Azim, Vibhu Sahni

**Affiliations:** ^1^Burke Neurological Institute, White Plains, New York 10605; ^2^Molecular Neurobiology Laboratory, Salk Institute for Biological Studies, La Jolla, California 92037; ^3^Weill Cornell Medicine, Feil Family Brain and Mind Research Institute, New York, New York 10065; ^4^Weill Cornell Graduate School of Medical Sciences, New York, New York 10065

**Keywords:** brainstem innervation, cerebellin, conditional AAV labeling, corticospinal, Crim1, Easi-CRISPR, genomic Cre reporters, segmental axon projection

## Abstract

The corticospinal tract (CST) facilitates skilled, precise movements, which necessitates that subcerebral projection neurons (SCPNs) establish segmentally specific connectivity with brainstem and spinal circuits. Developmental molecular delineation enables prospective identification of corticospinal neurons (CSNs) projecting to thoraco-lumbar spinal segments; however, it remains unclear whether other SCPN subpopulations in developing sensorimotor cortex can be prospectively identified in this manner. Such molecular tools could enable investigations of SCPN circuitry with precision and specificity. During development, Kelch-like 14 (*Klhl14*) is specifically expressed by a specific SCPN subpopulation, CSN_BC-lat_, that reside in lateral sensorimotor cortex with axonal projections exclusively to bulbar-cervical targets. In this study, we generated Klhl14-T2A-Cre knock-in mice to investigate SCPN that are *Klhl14+* during development into maturity. Using conditional anterograde and retrograde labeling in mice of either sex, we find that Klhl14-Cre is specifically expressed by CSN_BC-lat_ only at specific developmental time points. We establish conditional viral labeling in Klhl14-T2A-Cre mice as a new approach to reliably investigate CSN_BC-lat_ axon targeting and confirm that this identifies known molecular regulators of CSN axon targeting. Therefore, Klhl14-T2A-Cre mice can be used as a novel tool for identifying molecular regulators of CST axon guidance in a relatively high-throughput manner in vivo. Finally, we demonstrate that intersectional viral labeling enables precise targeting of only Klhl14-Cre+ CSN_BC-lat_ in the adult central nervous system. Together, our results establish that developmental molecular delineation of SCPN subpopulations can be used to selectively and specifically investigate their development, as well as anatomical and functional organization into maturity.

## Significance Statement

The cortex connects to brainstem and spinal targets through subcerebral projection neurons (SCPNs), which exhibit molecular diversity during development based on their neocortical location and axonal targets. We generated a novel Klhl14-Cre mouse line to utilize this developmental delineation and drive Cre expression in a specific SCPN subpopulation. This developmental specificity enabled investigation of (1) areal locations of Klhl14+ SCPN in mature cortex, (2) their axonal collateralization at maturity, and (3) which genes can control their axon targeting. Using intersectional tools, we can also selectively label these neurons in the adult CNS. Therefore, developmental molecular delineation of SCPN not only provides prospective identification but also enables molecular analysis during development, as well as anatomical and functional investigations in adulthood.

## Introduction

The corticospinal tract (CST) is a principal circuit responsible for skilled voluntary movements (Martin, 2005; Welniarz et al., 2017; Sahni et al., 2020). For such skilled motor control, it is critical that corticospinal neurons (CSN), which form a subset of all subcerebral projection neurons (SCPN), establish appropriate connectivity with their segmentally distinct targets. This necessitates that cortico-brainstem neurons project exclusively to the brainstem, while corticospinal projections extend to segmentally appropriate spinal targets in the cervical, thoracic, and lumbar cord. This segmental targeting specificity arises, in part, via axon extension specificity during development that is maintained into maturity (Sahni et al., 2021b). CSN subpopulations residing in distinct neocortical locations project to segmentally distinct targets (Sahni et al., 2021b). CSN in lateral cortex project axons exclusively to targets in the brainstem and cervical cord (CSN_BC-lat_). CSN in medial cortex (CSN_med_) are relatively more heterogeneous—a subpopulation extends axons exclusively to the brainstem and cervical cord (CSN_BC-med_) and resides interdigitated with another subpopulation that extends axons past these proximal targets to thoraco-lumbar segments (CSN_TL_).

These anatomically distinct subpopulations are also molecularly distinct during development (Sahni et al., 2021b). *Kelchlike-14* (*Klhl14*) is specifically expressed by CSN_BC-lat_, while *Cysteine rich transmembrane BMP regulator 1* (*Crim1*) is specifically expressed by CSN_TL_. We previously tested the hypothesis that developmental molecular delineation could prospectively identify these subpopulations into maturity. Using an inducible Crim1CreERT2 mouse line, we established that *Crim1* expression during development prospectively identified CSN_TL_ even before their differential axon targeting from other CSN subpopulations was evident. However, whether this developmental molecular delineation could enable identification and manipulation of other CSN subpopulations at maturity remained unknown. We therefore investigated whether CSN_BC-lat_ could similarly be identified during development via the expression of *Klhl14*. We generated Klhl14-T2A-Cre knock-in mice to label *Klhl14*+ SCPN during development and investigate their axonal projections at maturity. Our hypothesis was that SCPN, labeled via *Klhl14* expression during development, would only extend axons to bulbar-cervical segments at maturity. Surprisingly, however, we find that when we breed Klhl14-T2A-Cre mice with genomic Cre reporter mice, labeled SCPN axons extend to all levels of the neuraxis. Since this strategy labels all neurons that express *Klhl14* at any time during development, we next used a different approach to specifically label *Klhl14*+ SCPN at the developmental times when we had previously confirmed *Klhl14* expression specificity. Using conditional viral labeling, we find that Cre expression in Klhl14-T2A-Cre mice faithfully recapitulates this previously established specificity of *Klhl14* expression. Klhl14-Cre+ SCPN labeled at P0 reside in lateral sensorimotor cortex and extend axons exclusively to brainstem and cervical segments, i.e., *Klhl14* expression prospectively delineates CSN_BC-lat_. By comparing the axonal projections of *Klhl14*+ SCPN with all SCPN in lateral sensorimotor cortex, we establish that Cre expression in Klhl14-Cre mice labels nearly all CSN_BC-lat_. Our results highlight a limitation of using genomic Cre reporter mice when taking advantage of developmental molecular delineation to investigate CSN subpopulations into maturity.

We also establish the use of Klhl14-T2A-Cre mice as a tool to identify genes regulating CSN axon extension specificity during development. Our previous work had identified that misexpression of Crim1 (Sahni et al., 2021a) and Cbln1 (Song et al., 2023) in lateral sensorimotor cortex is sufficient to redirect CSN_BC-lat_ axons past their targets in the brainstem and cervical cord toward thoraco-lumbar spinal segments. In these investigations, we solely relied on the anatomical separation of CSN_BC-lat_ from CSN_med_. Klhl14-T2A-Cre mice enable selective labeling of CSN_BC-lat_ even in instances where AAV injections spread into medial cortex. Molecular delineation therefore provides a significant advance over using anatomical separation alone to target and investigate distinct CSN subpopulations. Finally, we describe the use of an intersectional viral labeling approach combining both Cre and Flp-dependent recombination to specifically target *Klhl14*+ CSN_BC-lat_ into maturity. Together, these results show that Cre expression in Klhl14-T2A-Cre at P0 labels all CSN_BC-lat_. More broadly, our results indicate that early developmental molecular delineation of distinct CSN subpopulations can be used to specifically label them prospectively with reliability and precision. These approaches can in turn enable investigating their anatomy and circuit-level function into maturity.

## Materials and Methods

### Generation of Klhl14-Cre mice

Klhl14-3xHA-T2A-iCre mice were generated to carry three HA-tags inserted at the C-terminus of Klhl14 protein followed by a T2A-iCre sequence. These mice were generated in the Transgenic Core Facility at the Salk Institute for Biological Studies, La Jolla, CA using the Easi-CRISPR strategy (Quadros et al., 2017). For this, long single-stranded DNA donors (ssDNAs, HDR template containing 3x HA tags-T2A-iCre flanked by 2 homology arms, 1,634 bp) were ordered from IDT (Megamer). These were injected into one-cell staged mouse zygotes along with two sgRNAs (made by Synthego) and Cas9 ribonucleoprotein (obtained from IDT) that together formed the CRISPR RNPs complex. The following two sgRNAs were used (1) Klhl14-sgRNA1: TGGATGGTGGCACTATGCCG; and (2) Klhl14-sgRNA2: ACCCTACAACAAATGACAGC.

For the microinjections, 3–4-week-old C57BL/6J donor female mice (Jackson Laboratory) were superovulated by 5 IU of PMSG and 5 IU of HCG injections and mated 1:1 with males. The next morning, one-cell stage embryos were collected and cultured in microdrops of KSOM + AA media (Millipore) covered with embryo-tested mineral oil at 37°C in 5% CO_2_. CRISPR RNP mixture was freshly prepared before microinjection by resuspending Cas9 protein (IDT), two sgRNAs (Synthego), and ssDNA (IDT) in microinjection buffer (10 mM Tris, pH 7.4–7.5, 0.15 mM EDTA) at a concentration of 20:10:10:5 ng/μl. CRISPR mix was injected into the male pronuclei using a Nikon Eclipse microscope and Narishige micromanipulators. Injected embryos were surgically transferred into the oviducts of 0.5 days post coitus (dpc) pseudopregnant CD1 females (Charles River Laboratories).

To confirm the correct insertion of the 3xHA-T2A-iCre into the Klhl14 locus, genomic DNA from the putative targeted pups were used for genotyping. The following primers were used to amplify the targeted insertion, which were located outside the ssDNAs (HDR template) in the Klhl14 locus:

1. Klhl14-gt-5a:GCAGAGGTCTTCCCAGTGTAGC; and
2. Klhl14-gt-3a: AGTTTGTGTAAAGGCGGAACAAAGC.

The resulting 1,703 bp PCR product was cloned and sequenced to confirm that it was correctly targeted into the Klhl14 locus. Targeted pups were bred with the wild-type mice to generate the first generation (F1) of the heterozygous mice. Once the Klhl14-3xHA-T2A-iCre mouse line was established, the following primers were used in a multiplex reaction for genotyping and to distinguish between Klhl14-Cre WT, Heterozygous and homozygous mice:

Klhl14-Cre (common forward): GAACCTGGACAGAACTCGA
Klhl14-WT Reverse: AGCCTGATATGGAAGAGTCTG
Klhl14-Cre Reverse: ACACAGACAGGAGCATCTTC

This reaction results in bands of the following sizes: WT allele, 334 bp; Klhl14-Cre allele, 467 bp.

### Mice

**Figure 1.**
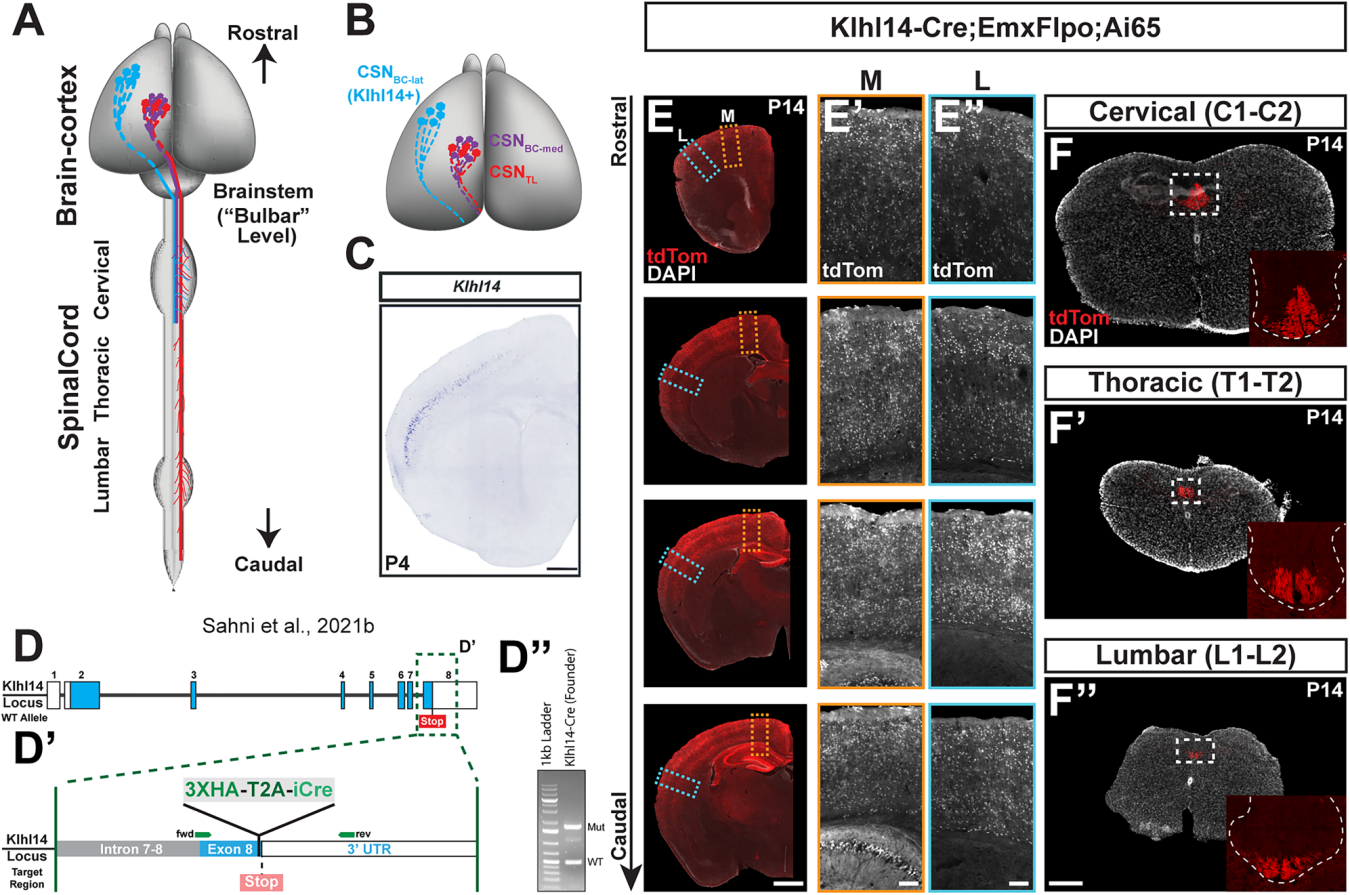
Genomic reporters of Cre-dependent recombination do not identify SCPN subpopulation specificity of Cre expression in Klhl14-Cre mice. ***A***, ***B***, Schematic (adapted from Sahni et al., 2021b) showing the locations of distinct CSN subpopulations and their corresponding axonal projections in mouse CNS. CSN_BC-lat_ (cyan) reside in rostrolateral cortex and extend axons only to bulbar-cervical targets. CSN_BC-med_ (purple) reside in medial cortex and extend axons only to bulbar-cervical targets. CSN_TL_ (red) reside in medial cortex and extend to thoraco-lumbar levels but extend collaterals across all spinal levels. *Klhl14* is only expressed by CSN_BC-lat_. ***C***, In situ hybridization image on a coronal section of a P4 mouse brain showing *Klhl14* expression by CSN_BC-lat_ (from Sahni et al., 2021b). ***D***, Schematic showing the genomic organization of the WT Klhl14 locus. Klhl14 is encoded by eight exons. ***D’***, Magnified view of region boxed in ***D*** showing the point of insertion of Cre in Klhl14-Cre mice. The T2A-Cre sequence was targeted to exon 8 immediately 3′ to the STOP codon. The remaining part of the 3′ UTR in exon 8 was used as a homology arm. ***D”***, Genomic PCR from F2 mice confirmed proper insertion of Cre, with a resultant product of 1,703 bp. ***E***, Coronal brain sections collected from a Klhl14-Cre;Emxflpo;Ai65 transgenic mouse. Klhl14-Cre-positive cell bodies are labeled via tdTomato reporter expression (in red and in monochrome). TdTomato+ neurons span all layers across both medial (***E’***) and lateral (***E”***) cortex. ***F***, Axial spinal sections from the same Klhl14-Cre;Emxflpo;Ai65 transgenic mouse taken at cervical, thoracic, and lumbar levels. TdTomato+ axons are present in the dorsal funiculus at all spinal levels consistent with the widespread recombination in cortex. Scale bars: 500 µm for ***C***, 1 mm for ***E***, 100 µm for ***E’***, ***E”***, and 250 µm for ***F***.

To generate mice for experimental analyses, we only used heterozygous Klhl14-Cre mice. For this, male Klhl4-Cre homozygous mice were mated with female CD-1 WT mice (Charles River Laboratories), and the resulting pups were used for both anterograde injections and retrograde injections. P0 was set as the day of birth. Both male and female mice were used in all analyses. The transgenic mouse used for Figure 1*E* resulted from mating a male Klhl14-Cre heterozygous mouse with female Emx1FlpO;Ai65 heterozygous mice. Emx1FlpO and Ai65 mice were maintained and genotyped as previously described (Madisen et al., 2015; Sahni et al., 2021b). Mice received food and water *ad libitum* and were housed on a 12 h on/off light cycle. All mouse studies were approved by the IACUC at Weill Cornell Medicine and the Salk Institute for Biological Studies, and all studies were performed in accordance with institutional and federal guidelines.

### Generation of AAV particles

The source, titers, and additional details for all AAVs used in this study are listed in Table 1. Constitutive and Cre-dependent AAV reporters were obtained either from Addgene or from the University of North Carolina Gene Therapy Center Vector Core.

**Table 1.** AAVs used in this study

AAV	Source	Catalog number	Titer
AAV2/1-hSyn-Crim1	Boston Children's Hospital Viral Core	N/A	5.67 × 10^13^ GC/ml
AAV2/1-hSyn-eGFP-2A-Cbln1	Boston Children's Hospital Viral Core	N/A	1.83 × 10^14^ GC/ml
AAV1-CAG-FLEX-tdTomato	UNC Gene Therapy Center Vector Core	N/A	5.9 × 10^12^ GC/ml
AAV1-hSyn-eGFP	Addgene	50465-AAV1	2.7 × 10^13^ GC/ml
AAV1-CAG-FLEX-eGFP-WPRE	Addgene	51502-AAV1	2 × 10^13^ GC/ml
AAV-CAG-tdTomato	Addgene	59462-AAV1	2 × 10^13^ GC/ml
rAAV-hSyn-Con/Fon-hChR2(H134R)-eYFP	Addgene	55645-AAVrg	1.2 × 10^13^ GC/ml
AAV1-EF1a-mCherry-IRES-Flpo	Addgene	55634-AAV1	1.3 × 10^13^ GC/ml
rAAV-FLEX-eGFP	Addgene	51502-AAVrg	2.3 × 10^13^ GC/ml
rAAV-tdTomato	Addgene	59462-AAVrg	2.5 × 10^13^ GC/ml

The table lists the source, titers, and additional details for all AAVs used in this study.

For generating AAVs to overexpress Crim1 and Cbln1, both genes were cloned into shuttle plasmids, where gene overexpression is driven by the human synapsin promoter (hSyn). For Cbln1, an eGFP-T2A coding sequence was placed in frame 3′ to the Cbln1 ORF. For AAV-Crim1, a GFP reporter could not be introduced since this exceeds the size limitations for AAV packaging. AAV particles were packaged by the Boston Children's Hospital Viral Vector Core.

### Ultrasound guided intracranial and intraspinal AAV injections

All AAV injections were performed in postnatal mice under visual guidance provided by ultrasound-mediated backscatter microscopy (Vevo 2100; VisualSonics) using previously described protocols (Sahni et al., 2021a,b; Song et al., 2023). For all injections, mouse pups were anesthetized using hypothermia, and injections were performed using a beveled glass micropipette attached to a nanojector (Nanoject III; Drummond Scientific). Following injections, pups were then placed on a heating pad and returned to their dams in home cages.

**Figure 2.**
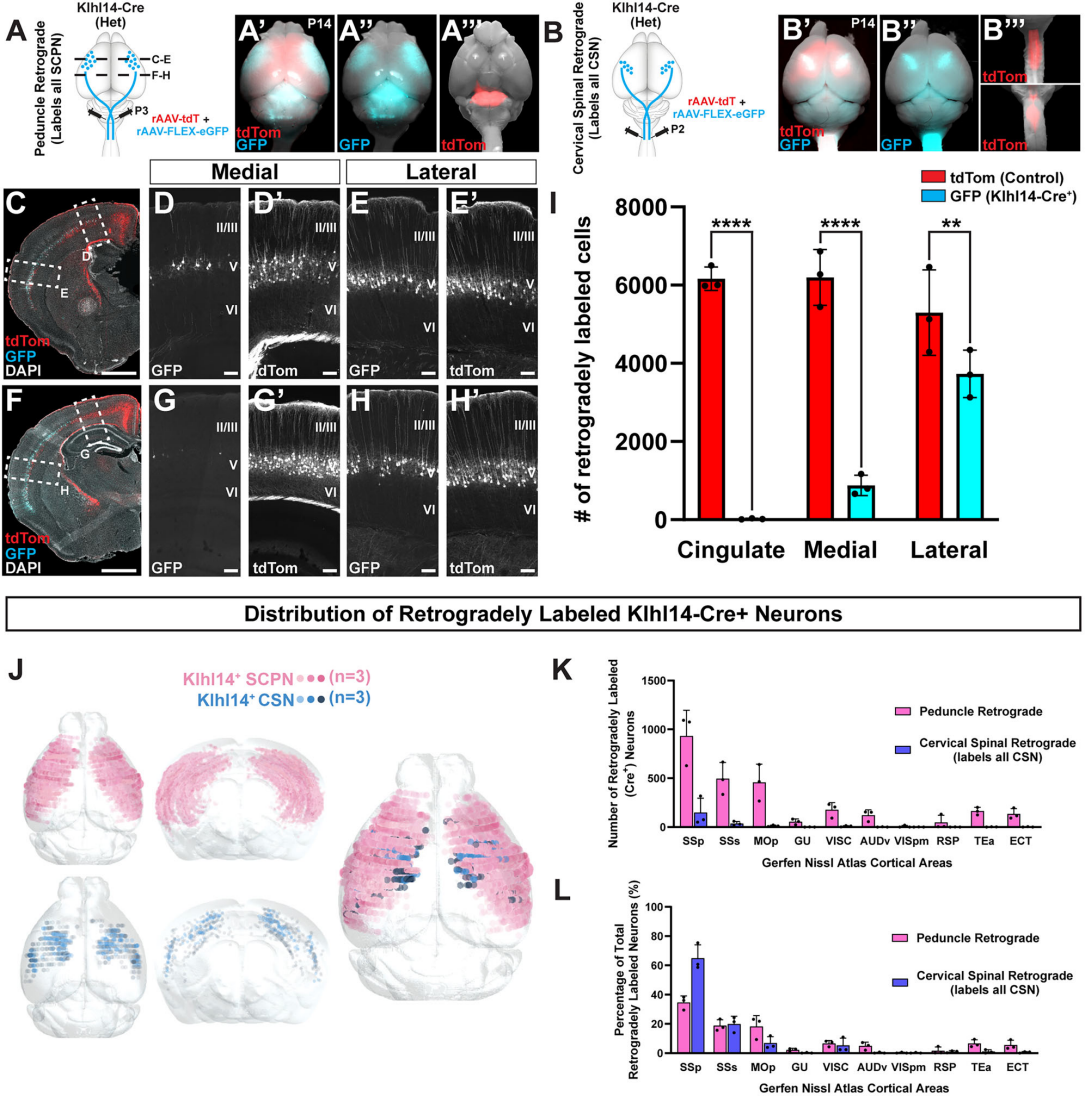
Conditional retrograde labeling identifies specificity of Klhl14-Cre expression by CSN_BC-lat_ enabling identification of their cortical location at maturity. ***A–A”’***, Klhl14-Cre heterozygous mice were coinjected with rAAV-FLEX-eGFP and rAAV-tdTomato in the cerebral peduncle at P3 (schematized in ***A***) and the brains collected at P14. ***A’***, ***A”***, Dorsal whole-mount view of the injected brain finds retrogradely labeled tdTomato^+^ SCPN span the entire sensorimotor cortex while eGFP^+^ (Klhl14-Cre+) SCPN occupy a smaller area, specifically residing in lateral cortex. ***A”’***, Dorsal (top) and ventral (bottom) whole-mount views reveal that the injection site, visualized via tdTomato expression, is restricted to the level of the rostral pons (cerebral peduncle) and does not extend into cervical spinal cord. ***B–B”’***, Klhl14-Cre heterozygous mice were coinjected with rAAV-FLEX-eGFP and rAAV-tdTomato in cervical spinal cord at P2 (schematized in ***B***) and brains collected at P14. ***B’***, ***B”***, Whole-mount view of the injected brains finds fewer tdTomato+ CSN labeled than the overall population of SCPN in ***A”***. eGFP^+^ CSN occupy a smaller area of cortex than tdTomato^+^ CSN, indicating specificity of Cre expression. ***B”’***, Dorsal (top) and ventral (bottom) whole-mount views reveal that the injection site, visualized via tdTomato expression, is restricted to the cervical spinal cord and does not extend rostrally into the pons. ***C–H’***, Coronal sections of the same brain in ***A*** at two distinct rostrocaudal levels reveal that tdTomato^+^ SCPN reside in both medial and lateral cortex (magnified views in ***D’***, ***G’***, ***E’***, ***H’***). eGFP^+^ SCPN mostly reside in lateral (***E***, ***H***) and not medial (***D***, ***G***) cortex, which is consistent with previously established expression of *Klhl14*. ***I***, Quantification of retrogradely labeled tdTomato+ versus eGFP+ SCPN (*n* = 3 mice). Two-way ANOVA: cortical area × Cre status *F*_(2,12)_ = 24.02, *p* < 0.0001; cortical region *F*_(2,12)_ = 8.526, *p* =0.0050; genotype/Cre status *F*_(1,12)_ = 228.4, *p* < 0.0001. Tukey's multiple-comparison posttest shows that there is a significant difference in the number of tdTomato+ versus eGFP+ (Klhl14-Cre+) SCPN in all three cortical regions (****p < 0.0001 in cingulate cortex, ****p < 0.0001 in medial cortex, and **p = 0.0084 in lateral cortex). The significant difference present in lateral cortex implies the existence of another subpopulation of lateral SCPN negative for *Klhl14*. The number of eGFP+ SCPN in lateral cortex is also significantly higher than in both cingulate (*p* < 0.0001) and medial (*p* < 0.0003) cortex. This confirms that the vast majority of eGFP^+^ SCPN (>80%) reside in lateral cortex. There is no significant difference in the number of control tdTomato+ SCPN across the three cortical regions. ***J***, 3D AMaSiNe reconstructions of conditionally labeled neurons from three Klhl14-Cre mice each that received retrograde labeling from either the cerebral peduncle (Klhl14-Cre+ SCPN, pink) or cervical spinal cord (Klhl14-Cre+ CSN, blue). SCPNs occupy a much larger area of cortex compared with CSN, but both primarily reside in lateral cortex. ***K***, Distribution of Klhl14^+^ SCPN across different cortical areas (annotations as assigned by the Allen Brain Atlas). Most Klhl14-Cre+ neurons reside in somatosensory cortices. ***L***, Percentage counts of labeled Klhl14-Cre+ neurons in each area, again demonstrating that the majority of Klhl14+ SCPN and CSN reside in sensory cortices. Scale bars: 1 mm for ***C***, ***F***, and 100 µm for ***D***, ***E***, ***G***, ***H***.

For intracortical injections for anterograde labeling, the micropipette was inserted unilaterally into rostrolateral cortex using defined landmarks. Mice received two sets of 15 injections of 23 nl each: one each at two different rostrocaudal levels. Injections were performed at 1 s intervals, with a 10 s waiting period after the 15th injection before the micropipette was removed. Retrograde labeling from the cerebral peduncles was performed at P0, while labeling from the cervical cord was performed at postnatal day (P) 2 using established protocols (Sahni et al., 2021a,b; Song et al., 2023). For labeling from the cerebral peduncle, AAV injections were delivered in the rostral pons as a set of seven injections of 23 nl each, at three different injection sites per hemisphere. For labeling from the cervical cord, we injected 10 injections of 23 nl each on either side of the midline. For retrograde labeling experiments shown in Figure 2, from either the cerebral peduncles or cervical dorsal funiculus, identical rAAVs were used.

**Figure 3.**
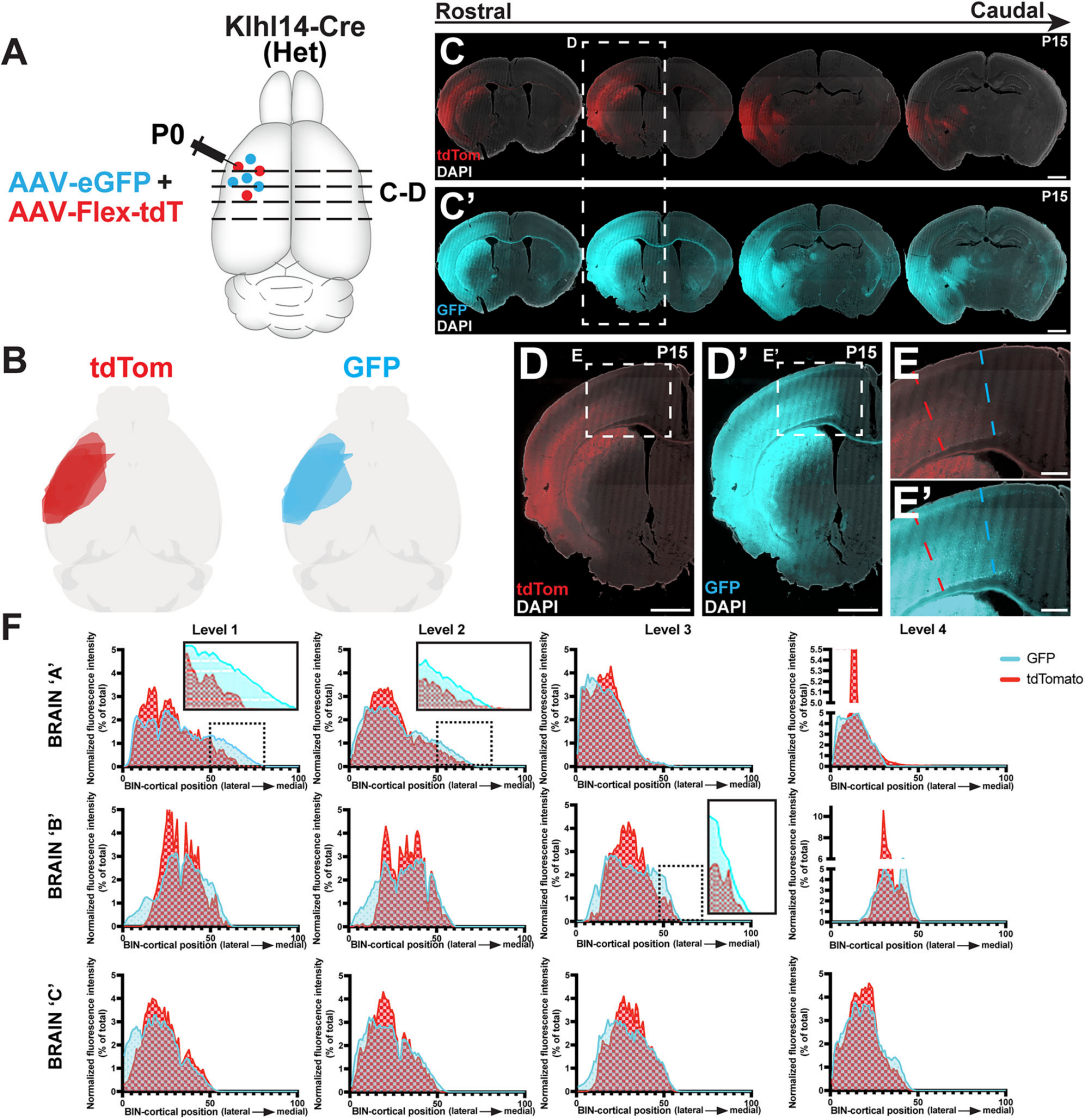
Anterograde labeling in Klhl14-T2A-Cre mice confirms that Klhl14+ SCPN reside in lateral but not medial cortex. ***A***, Schematic of experimental outline: Klhl14-Cre heterozygous mice were coinjected with AAV-eGFP and AAV-FLEX-tdTomato at P0 in rostrolateral cortex, where Klhl14-Cre+ SCPN reside. Samples were collected at P15. ***B***, Overlaid 3D reconstructions of cortical volumes of both eGFP and tdTomato reporters from seven different injected mice. Note that the eGFP (non-Cre dependent) reporter expression spans more medially than tdTomato (Cre-dependent). ***C***, ***C’***, Series of coronal brain sections from an injected mouse spanning rostral (left) to caudal (right) showing spread of tdTomato (***C***) and eGFP (***C’***) reporters in cortex. ***D***, ***D’***, Zoomed in images for the section marked by dotted outline in ***C*** showing tdTomato (***D***) and eGFP (***D’***) fluorescence. ***E’***, ***E”***, Zoomed in images for the area marked by dotted outlines in ***D***, ***D’***. Red dotted lines indicate the medial most point of tdTomato fluorescence, while cyan dotted lines indicate the medial most extent of eGFP fluorescence. Note that the cyan lines are present more medially than the red lines. ***F***, Fluorescence intensity plots for both tdTomato (red) and eGFP (cyan) in cortical layer V from three separate mice across the same four cortical levels as in ***C***. Each individual plot shows the analysis from one coronal brain section of a given mouse. Areas of eGFP and tdTomato fluorescence largely overlap; however, eGFP spreads more medially in two of three mice—levels 1 and 2 in Brain “A” and level 3 in Brain “B” (insets highlight the specific sections in the individual plots where medial spread occurs in those three sections from the two mice). There is no medial spread in Brain C. This higher variability of eGFP compared with FLEX-tdtom despite coinjection demonstrates the efficacy of Klhl14-Cre to specifically target CSN_BC-lat_. Scale bars: 1 mm for ***C***, ***D*** and 250 µm for ***E’***, ***E”***.

**Figure 4.**
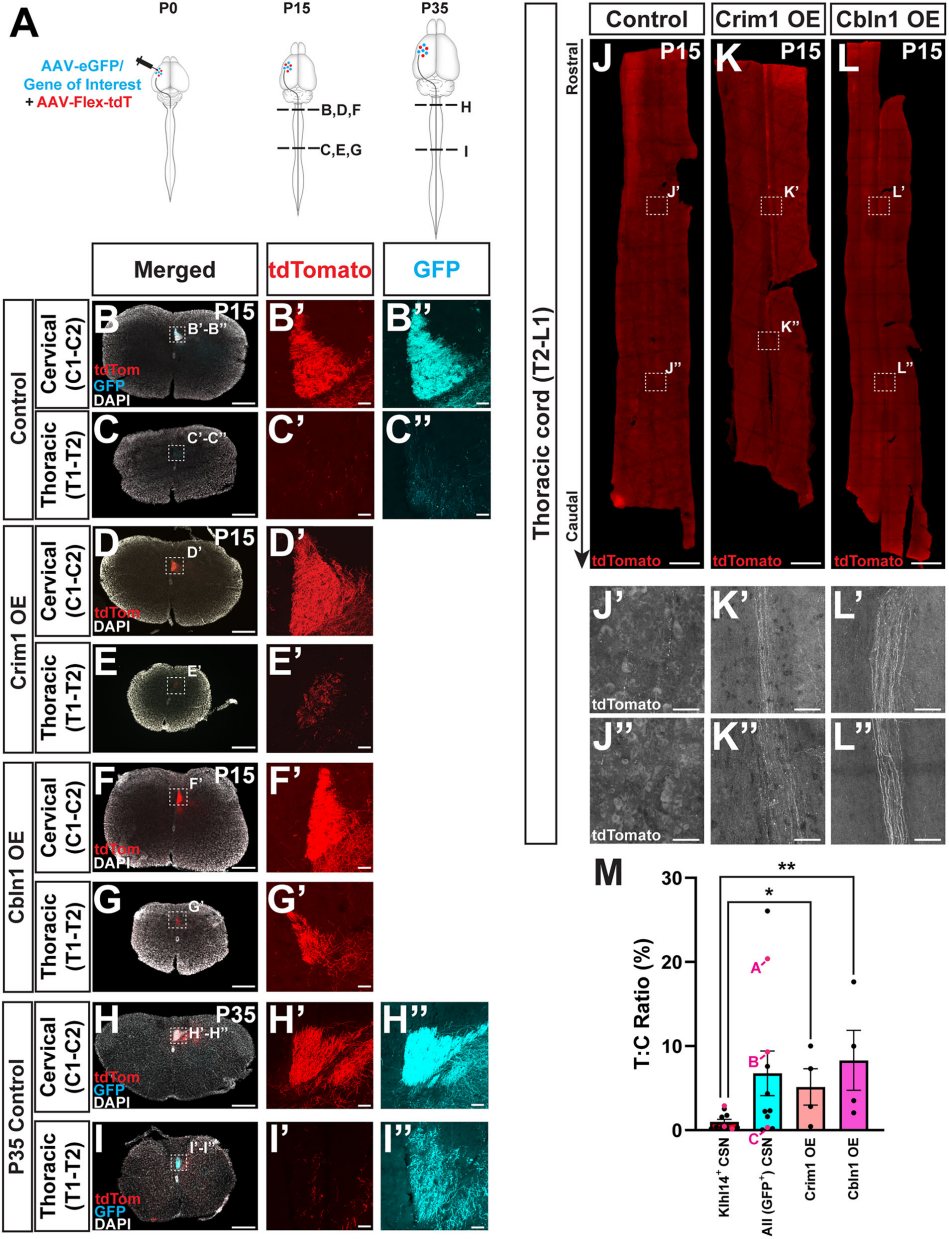
Misexpression of Crim1 and Cbln1 in Klhl14-T2A-Cre mice redirects Klhl14+ CSN axons into thoracic cord. ***A***, AAV injection and sample collection is identical to Figure 3. P0 Klhl14-Cre heterozygous mice were coinjected into lateral cortex with AAV-FLEX-tdTomato and either one of following three AAVs (1) AAV-eGFP (control, as shown in Fig. 3); (2) AAV-Crim1(Crim1 OE); or (3) AAV-Cbln1 (Cbln1 OE). Tissue was analyzed at P15. In addition, and different from Figure 3, three mice that received control AAV-eGFP were perfused at P35. Images from these mice are shown in ***H–I”***. All remaining images and quantification are from mice that were perfused at P15. ***B***, ***C***, Spinal axial sections from an injected Klhl14-Cre mouse at P15 at cervical C1–C2 (***B–B”***) and thoracic T1–T2 (***C–C”***) levels. While both tdTomato+ (***B’***) and eGFP+ axons (***B”***) are seen in the cervical dorsal funiculus, tdTomato+ axons (***C’***) are not present in thoracic spinal cord. AAV-eGFP-labeled axons (***C”****)* are present in the thoracic spinal cord, likely due to labeling of CSN_TL_. ***D–G***, Spinal axial sections from a Klhl14-Cre mouse at P15 that was injected with either Crim1 OE (***D***, ***E***) or Cbln1 OE (***F***, ***G***). In both mice, Klhl14-Cre+ CSN axons extend to the thoracic dorsal funiculus (***E***, ***E’***, ***G***, ***G’***), consistent with previously known functions for each gene. ***H***, ***I***, Spinal axial sections from a P35 Klhl14-Cre mouse that received intracortical injections with AAV-FLEX-tdTomato and AAV-eGFP at P0. Similar to results at P15, there is lack of axon extension by Klhl14-Cre+ CSN axons to thoracic segments (similar to ***C’***), while a significant number of eGFP+ axons are present in the thoracic dorsal funiculus (similar to ***C”***). ***J–L***, Maximum intensity projections of horizontal sections of the thoracic cord (T2–T13) from control (***J***), Crim1 OE (***K***), and Cbln1 OE (***L***) mice. Monochrome magnified views of rostral and caudal levels from each cord are shown in ***J’–L”***. In control mice, no CST axons are present within the first few thoracic segments (***J’***). In contrast, Klhl14-Cre+ CSNc axons in both Crim1 OE (***K”***) and Cbln1 OE (***L”***) mice extend to far caudal levels in the thoracic cord. ***M***, CST intensity was quantified in three axial sections per spinal level (cervical C1–C2, thoracic T1–T2) per mouse, and the mean value across three axial sections is plotted. The corresponding T:C ratios are shown for both eGFP+ as well as tdTomato+ axons in control mice (*n* = 11). There is greater variability in the T:C ratio for eGFP+ axons than tdTomato+ axons. The T:C ratios of both eGFP+ and tdTomato+ axons from the three control mice analyzed for fluorescence intensity in neocortical layer V in Figure 3*F* are labeled as “A,” “B,” and “C” in the graph. Graph also shows T:C ratios for tdTomato+ axons from Crim1 OE (*n* = 4) and Cbln1 OE (*n* = 4) mice. Overexpression of either Crim1 or Cbln1 results in a significant increase in percentage of Klhl14-Cre+ CSN axons that extend into thoracic cord compared with control mice [statistical analysis done using Kruskal–Wallis test; *H*_(2)_ = 10.18, *p* = 0.0022, followed by a post hoc Mann–Whitney *U* test for each pairwise comparison (*p = 0.0264 for control vs Crim1 OE; **p = 0.0059 for control vs Cbln1 OE)]. Scale bars: 250 µm for ***B–I***, 20 µm for insets in ***B’–I”***, 500 µm for ***J–L***, and 100 µm for ***J’***, ***J”***, ***K’***, ***K”***, and ***L’***, ***L”***.

**Figure 5.**
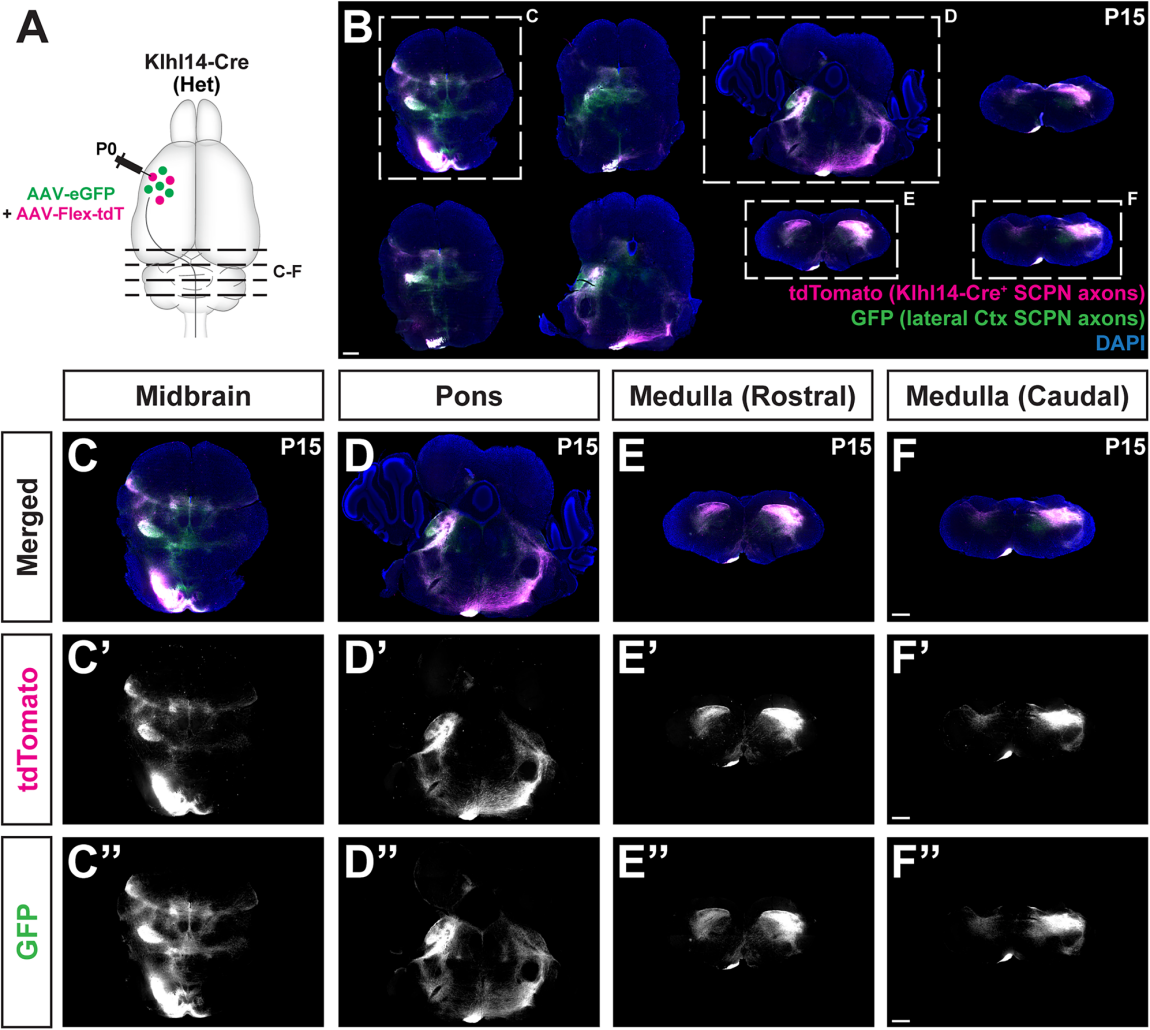
Klhl14+ SCPN axons exhibit identical projections in the brainstem as anatomically labeled CSN_BC-lat_. ***A***, Similar experimental outline as in Figure 3. Klhl14-Cre knock-in mice were coinjected with AAV-eGFP and AAV-FLEX-tdTomato into rostrolateral cortex at P0. eGFP+ axons are from CSN_BC-lat_. The brainstem from injected mice was analyzed at P15. ***B***, Coronal sections of the brainstem from one such injected mouse spanning the rostrocaudal extent of the brainstem showing axonal collateralization by Klhl14-Cre+ SCPN (magenta) versus anatomically defined eGFP+ CSN_BC-lat_ (green). ***C–F***, Magnified view of sections highlighted in ***B*** showing midbrain (***C***), pons (***D***), rostral medulla (***E***), and caudal medulla (***F***). Klhl14-Cre+ SCPN axons exhibit nearly identical collateralization across the brainstem as eGFP+ CSN_BC-lat_ axons. Scale bars: 500 µm.

rAAV injections either at the level of the cerebral peduncles or in the cervical dorsal funiculus cover the whole extent of the fiber tracts to retrogradely label any neuron that extends to that level. This is distinct from injection paradigms that are restricted to the gray matter and interrogate specificity of innervation at a given spinal segment (further discussed in Sahni et al., 2021b). The injection into the cervical dorsal funiculus therefore labels all corticospinal neurons, including those that project to more caudal spinal segments, which is evidenced by the widespread labeling within primary motor cortex (in medial cortex) spanning cortical areas with lumbar-projecting neurons as previously described (Sahni et al., 2021b). Further, retrograde labeling from the cerebral peduncle labels nearly four times as many control tdTomato+ neurons than a similar injection into the cervical dorsal funiculus (data not shown). In all experiments using coinjections of different AAVs (in Figs. 2–5), the two AAVs used had identical titers (as indicated in Table 1). The AAVs were loaded in a 1:1 volumetric ratio into one micropipette and coinjected simultaneously.

**Figure 6.**
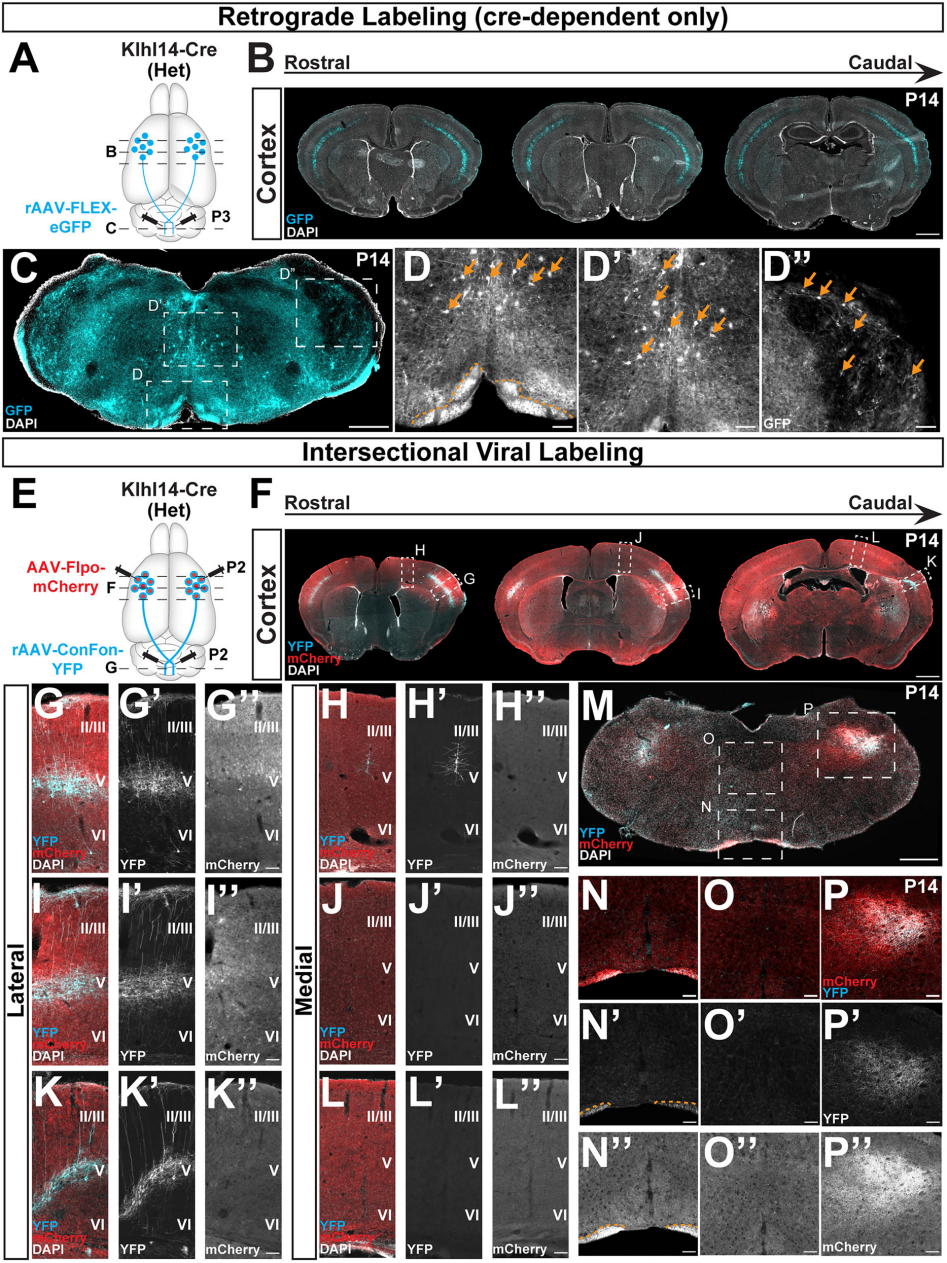
An intersectional approach to target Klhl14+ SCPN at maturity using Klhl14-Cre mice. ***A***, Schematic of experimental outline: Klhl14-Cre mice received a bilateral retrograde injection of a retro AAV-FLEX-eGFP into the cerebral peduncle at P3. ***B***, Coronal brain sections from an injected mouse at P14 show eGFP+ (Klhl14-Cre+) SCPN in lateral sensorimotor cortex. ***C***, A representative section from the medulla from an injected mouse shows numerous eGFP+ neurons labeled at medial (***D***, ***D’***) and lateral (***D”***) levels. Retrograde injection of AAV-FLEX-GFP therefore also labels Klhl14-Cre+ neurons in the brainstem. ***E***, Schematic of experimental outline: Klhl14-Cre mice received bilateral intracortical injections of AAV-Flpo-mCherry into rostrolateral cortex at P2 along with bilateral retrograde injections of AAV-ConFon-eYFP in the cerebral peduncle. ***F***, Coronal brain sections from a P14 injected mouse showing AAV-Flpo-mCherry (red) in lateral cortex. YFP+ (Cre+ and FlpO+) SCPN, i.e., Klhl14-Cre+ SCPN are specifically labeled in lateral cortex. ***G–L***, Zoomed in images of lateral (***G***, ***I***, ***K***) and medial (***H***, ***J***, ***L***) cortex for the same sections shown in ***F*** (demarcated by dotted outlines). While mCherry labeling is present in multiple cortical layers in lateral cortex (***G”***, ***I”***, ***K”***), YFP+ neurons are restricted to layer V primarily in lateral cortex (***G’***, ***I’***, ***K’***). ***M***, Representative coronal section of the medulla from the same mouse. ***N–P***, Zoomed in images of the regions demarcated in ***M***. Yellow dotted outlines in ***N’***, ***N”*** demarcate the pyramidal tract traversed by CSN_BC-lat_ axons that are both YFP+ (***N’***) and mCherry+ (***N”***). We find YFP+ (Klhl14-Cre+) SCPN axons (***P’***), but no cell bodies are YFP+ (***N’***, ***O’***). YFP+ and mCherry+ axon collaterals occupy similar territories (***P***, ***P’***, ***P”***). Therefore, this intersectional viral labeling approach labels Klhl14-Cre+ SCPN in cortex but avoids labeling of Klhl14-Cre+ neurons in the brainstem, highlighting the effectiveness of this intersectional strategy for only targeting Klhl14-Cre+ SCPN at maturity. Scale bars: 1 mm in ***B***, ***F***, 500 µm for ***C***, ***M***, and 100 µm for ***G–L”***, ***N–P”***.

For intersectional viral labeling in Figure 6, mice were either injected at P3 with rAAV-FLEX-eGFP or at P2 with rAAV-Cre-On-Flp-On-eYFP bilaterally into the cerebral peduncle. On the same day, mice which received the Cre-On-Flp-On-eYFP also received intracortical injections with AAV-mCherry-Flpo into rostrolateral cortex. For these injections, mice received one set of 15 injections (per cerebral hemisphere) of AAV-mCherry-Flpo at 23 nl per injection.

### Tissue collection and preparation

P14 or P15 mice were killed using an intraperitoneal injection of 150 mg/kg ketamine along with 15 mg/kg xylazine. Mice were transcardially perfused, first with 1× PBS, followed by 4% paraformaldehyde (PFA). The brain and spinal cord were dissected and postfixed in 4% PFA overnight at 4°C. Samples were rinsed with 1× PBS and stored in PBS-azide (0.025% sodium azide in 1× PBS) at 4°C for long-term storage. For downstream processing, samples were first cryoprotected in sucrose PBS (30% sucrose in 1× PBS), frozen in Tissue-Tek OCT compound (Sakura Finetek), and cryosectioned on a Leica CM3050 S cryostat (Leica Microsystems). For analyzing the cortex, prior to freezing, the cerebellum, pons, and medulla were removed as one tissue block from the forebrain using a razor blade. Then, 50 μm coronal brain sections were collected in PBS-azide. The brainstem samples were then frozen separately and serial sections collected in PBS-azide. For axon quantification analysis on axial spinal sections, 50 µm axial sections from C1–C2 and T1–T2 spinal cord were collected in PBS-azide. For horizontal sections shown in Figure 5, the thoracic cord was separated from the lumbar cord at T13/L1 using a razor blade. An orientation cut was made at the caudal end prior to freezing to identify the rostral and caudal ends of the cord in horizontal sections. Then, 70-µm-thick horizontal sections were collected serially in 1× PBS.

### Immunohistochemistry

Immunohistochemistry was performed as previously described (Sahni et al., 2021a,b; Song et al., 2023). Sections were blocked for 30 min at room temperature in 1× PBS containing 0.3% BSA and 0.3% Triton X-100, followed by overnight incubation at 4°C with the primary antibody, which was diluted in the same blocking solution. Following three rinses in 1× PBS, sections were incubated for 3 h at room temperature in secondary antibodies (1:750) in the same blocking solution. After three rinses in 1× PBS, sections were mounted onto glass slides. Coverslips were affixed using DAPI Fluoromount-G (SouthernBiotech, 0100-20) and sealed using clear nail polish. Slides were then allowed to air-dry overnight before being imaged. The following primary antibodies were used: Rabbit anti-RFP (1:750; Rockland Immunochemicals) for analyzing tdTomato+ axons in the brainstem and spinal cord, Rabbit anti-GFP (1:500; Thermo Fisher Scientific, A-11122) for analyzing both eGFP and YFP fluorescence in the brainstem in Figure 6. The following secondary antibodies were used: Alexa Fluor 546 goat-anti-rabbit (Thermo Fisher Scientific, A-11035), Alexa Fluor 488 goat-anti-rabbit (Thermo Fisher Scientific, A-11008). Coronal forebrain (cortical) sections did not undergo immunohistochemical processing. Free-floating sections were mounted on glass slides and coverslipped as above.

### Microscopy and imaging

Prior to tissue sectioning, all brains were first whole-mount imaged (dorsal view) on a Nikon SMZ18 stereomicroscope (Nikon). Epifluorescence images were acquired on a Zeiss Axio Imager M2 microscope (Zeiss) using Stereo Investigator Software (MBF Bioscience). For axon extension quantification on axial spinal sections, labeled axons in the dorsal funiculus were imaged on a Leica SP8 confocal microscope (Leica Microsystems) with 63× oil immersion lens using LASX software (Leica Microsystems). Confocal images of horizontal sections of the thoracic cord were acquired at 20×, and maximum intensity projections were produced using Fiji ImageJ (National Institutes of Health).

### Three-dimensional injection volume reconstruction and quantification of AAV fluorescence in layer V

To reconstruct and quantify three-dimensional (3D) injection volumes of tdTomato+ and eGFP+ neurons within cortex, we used the open-source VOL3D pipeline (https://github.com/jkaiser87/VOL3D). This pipeline transfers 2D injection outlines from coronal brain sections into the Allen Mouse Brain Common Coordinate Framework version 3 (CCFv3, 10 µm atlas; Wang et al., 2020). Briefly, injection areas were first manually outlined in coronal sections using FIJI/ImageJ (version 1.54f; Schindelin et al., 2012). Outlines were then transferred by applying affine transformations (based on the pipeline AP_histology; https://github.com/petersaj/AP_histology; 2019/2024; Peters, 2024) to register coordinates into CCFv3 space. Within VOL3D, surface boundaries of the injection volumes were computed via Delaunay triangulation and Laplacian smoothing to generate 3D representations within the CCFv3 reference brain. To quantify the spread of eGFP versus tdTomato fluorescence in layer V, we modified our previously used protocol (Sahni et al., 2021a). Briefly, matched coronal 50-μm-thick sections at the four different rostrocaudal levels from the three different mice were imaged for eGFP and tdTomato. Layer V was identified using DAPI and the entire layer V from pyriform cortex (lateral end) to cingulate cortex (medial end) was first cropped in Adobe Photoshop and then straightened using the “Puppet Warp” function. The resulting rectangular image was binned into 100 segments, using the divide slice function in Adobe Photoshop, with the lateral most point placed at “0.” Fluorescence intensity of both eGFP and tdTomato in each bin was quantified using the measure function in Fiji (NIH). The fluorescence value for each channel (eGFP and tdTomato) in each bin was normalized to the total fluorescence measured for that channel in that section. These normalized values are plotted for the mediolateral position (for each bin) in Figure 3*F*.

### Quantification and spatial representation of retrogradely labeled neurons

To generate 3D spatial maps of retrogradely labeled Klhl14-Cre+ SCPN/CSN, we used the publicly available AMaSiNe pipeline (Song et al., 2020; https://github.com/vsnnlab/AMaSiNe) following the standard protocol. Quantification of labeled neurons in each cortical area (e.g., MOp, MOs) was performed using NeuroInfo (MBF Bioscience), based on automated anatomical assignments using the Allen Mouse Brain Atlas. To assess the medial-lateral distribution of labeled neurons, we used CELL3D (https://github.com/jkaiser87/CELL3D; Kaiser, 2024), a publicly available spatial analysis pipeline. Each cortical hemisphere was divided into five equal-width bins along the medial-lateral axis, which was performed individually for each coronal section. The most medial bin was assigned as cingulate cortex, the next two bins as medial, and the outer two bins as lateral cortex, following previously described criteria (Sahni et al., 2021b). This classification revealed that Klhl14-Cre+ SCPN are predominantly located in lateral cortex, corresponding to somatosensory regions (Fig. 2*K*), consistent with prior descriptions of Klhl14 expression in CSNBC-lat neurons (Sahni et al., 2021a,b).

### Axon extension quantification

For axon extension analysis in the spinal cord, we only included mice in which there was detectable tdTomato fluorescence in the whole-mount images. For quantification, confocal images were analyzed using a custom-made macro in Fiji. Briefly, all confocal *Z*-stacks were merged to create a maximum projection image. The dorsal funiculus was manually annotated as a region of interest (ROI) in this image. The ROI was thresholded in the three brightest *Z*-planes to create a binary image, and the area of thresholded pixels was summed. For each animal, three axial sections were analyzed at each spinal level (cervical and thoracic) and the area averaged. For each animal, the average area at thoracic T1–T2 was divided by the average area at cervical C1–C2 to obtain the T:C ratio, which was expressed as a percentage.

### Statistical analyses

Statistical analysis was performed in Prism 10.4 (GraphPad Software). For comparison between retrogradely labeled SCPN in cingulate, medial, and lateral cortex in Figure 2, we used a two-way ANOVA followed by a Tukey's multiple-comparison test. For comparison between the T/C ratios in Figure 4, we used the Kruskal–Wallis test, followed by a post hoc unpaired Mann–Whitney *U* test for comparison between individual groups. Data are presented as mean ± SEM (standard error of mean), with *n* indicating the number of mice used in each group for comparison. Male and female mice were used without distinction in experiments.

### Code accessibility

The following analysis pipelines were used in this study:

1. VOL3D for 3D injection volume reconstruction (https://github.com/jkaiser87/VOL3D; Kaiser, 2024)
2. CELL3D for medial-lateral classification of cortical neurons (https://github.com/jkaiser87/CELL3D; Kaiser, 2024)
3. AMaSiNe for 3D spatial mapping of retrogradely labeled neurons (https://github.com/vsnnlab/AMaSiNe; Song et al., 2020)

All tools are freely available under open-source licenses at the URLs provided.

## Results

### Genomic Cre reporter mice show that Klhl14-T2A-Cre drives widespread recombination across the neocortex

Our previous work has established that CSN in lateral cortex, i.e., CSN_BC-lat_, are relatively more homogeneous than CSN in medial cortex, whereby CSN_BC-lat_ extend axons exclusively to bulbar-cervical segments (schematized in Fig. 1*A*,*B*; Sahni et al., 2021b). We had established *Klhl14* expression using in situ hybridization. In the developing neocortex, from embryonic day (E) 18.5 to P7, *Klhl14* is expressed by SCPN in lateral, but not medial, layer V, i.e., *Klhl14* is specifically expressed by CSN_BC-lat_ (Fig. 1*C*). However, these analyses could not investigate the axonal trajectory of *Klhl14*-expressing neurons during development at maturity. Therefore, to establish this, we generated Klhl14-T2A-Cre knock-in reporter mice (hereby referred to as “Klhl14-Cre”), in which we introduced a 3xHA-T2A-iCre cassette in frame with the Klhl14 coding sequence immediately 5′ to the STOP codon (Fig. 1*D*,*D”*). Proper insertion of the Cre coding sequence within the Klhl14 locus was confirmed by PCR (Fig. 1*D”*) followed by sequencing.

We next used these mice to investigate axonal projections of *Klhl14*+ SCPN. In these experiments, we were specifically interested in Klhl14-Cre expression by neocortical SCPN. However, since *Klhl14* is expressed by a subpopulation of spinal interneurons, we therefore also used Emx1-IRES-FlpO to drive FlpO recombinase expression exclusively in neocortical projection neurons, with no spinal expression. We crossed Klhl14-Cre mice with Emx-IRES-FlpO (Sahni et al., 2021b) and Ai65 (Madisen et al., 2015) reporter mice, to generate Klhl14T2A-Cre;Emx1-IRES-FlpO;Ai65 triple transgenic mice. In Ai65 reporter mice, tdTomato expression only occurs upon both Cre- and FlpO-mediated recombination. This breeding strategy therefore enabled us to avoid labeling any spinal *Klhl14*-expressing cells and specifically investigate tdTomato+ axonal projections of *Klhl14*+ SCPN. Our prediction was that only CSN_BC-lat_ would be tdTomato+ in these mice. However, we find that tdTomato+ neurons span all cortical layers and not just layer V and also reside across the entire mediolateral axis (Fig. 1*E–E”*). Furthermore, and in line with the distribution of tdTomato+ neurons in cortex, we find that tdTomato+ SCPN axons extend beyond the cervical cord to thoracic and lumbar levels (Fig. 1*F–F”*). These results would suggest that Cre expression in Klhl14-Cre mice does not recapitulate the known expression of *Klhl14*. However, because this breeding strategy with genomic Cre reporter mice does not allow for temporal control over the timing of recombination, it remained possible that Cre expression in Khl14-Cre mice occurred correctly at the appropriate developmental stages. We therefore tested this possibility via conditional anterograde and retrograde labeling in Klhl14-Cre mice at these developmental stages using AAV reporters.

### Conditional retrograde labeling identifies specificity of Cre expression by CSN_BC-lat_ and cortical location of CSN_BC-lat_ at maturity

One possible explanation for the broad Cre reporter expression when using genomic Cre reporter mice is that *Klhl14* is expressed more broadly, prior to P0, in the embryonic neocortex. This would in turn lead to more widespread Cre-mediated recombination across sensorimotor cortex. In prior work, we had used in situ hybridization to establish that there is some embryonic *Klhl14* expression in neocortex (Sahni et al., 2021b). While there was no detectable expression at E15.5, we had observed expression at E18.5, which was restricted to lateral cortex. This was therefore still strikingly different from the widespread expression seen in the triple transgenic intersectional genetic reporter mice at P0 shown in Figure 1. This would suggest that this reporter expression was being driven by broader embryonic expression that occurred before E15.5. Another possibility is that Cre expression in Klhl14-Cre mice is being driven by other genomic regulatory elements and therefore, as a result, Cre expression does not reflect *Klhl14* expression. This latter possibility would indicate Klhl14-Cre mice cannot be used to investigate any *Klhl14*+ neuronal populations. To distinguish between these two possibilities, we used retrograde AAVs (rAAV; Tervo et al., 2016) to conditionally label all Cre+ SCPN in Klhl14-Cre mice at P0, i.e., past embryonic developmental stages and at a time when we had previously identified Klhl14 expression specificity (Sahni et al., 2021a,b). We used established protocols to inject rAAVs into the axon tracts, i.e., directly into the white matter to label all neurons that extend axons to that level of the neuraxis.

We coinjected two rAAVs into the cerebral peduncle of Klhl14-Cre mice at P3: one constitutively expressing tdTomato (rAAV-CAG-tdTomato) and one that conditionally expressed eGFP upon Cre expression (rAAV-FLEX-eGFP; Fig. 2*A*). A ventral view of the injection site, labeled via tdTomato expression, is shown in Figure 2*A”’* highlighting that rAAV labeling remains restricted to the level of injection. If Cre expression in Klhl14-Cre mice faithfully reflected our previously established *Klhl14* expression (as shown in Fig. 1*C*; Sahni et al., 2021a,b), we would predict that eGFP+ SCPN would be restricted to lateral but not medial cortex. However, if Cre expression does not reflect *Klhl14*, then eGFP+ SCPN would be present across both medial and lateral sensorimotor cortex, similar to our results obtained using genomic Cre reporter mice shown in Figure 1*E*. We analyzed injected mice at P14. Whole-mount views in Figure 2*A’*,*A”* show that while tdTomato expression spans the entire sensorimotor cortex, eGFP fluorescence is restricted to lateral cortex (Fig. 2*A”*). Analysis of coronal sections from labeled mice at two different rostrocaudal levels (Fig. 2*C*,*F*) shows that while SCPN across both medial and lateral cortex are tdTomato+ (Fig. 2*D’*,*G’*,*E’*,*H’*), eGFP+ SCPNs are largely restricted to lateral sensorimotor cortex (Fig. 2*D*,*G*,*E*,*H*). Quantification of number of retrogradely labeled SCPN from three different injected mice shows that there is no difference in the numbers of tdTomato+ SCPN in cingulate, medial, and lateral cortex. Strikingly, >80% of eGFP+ SCPNs are in lateral cortex (Fig. 2*I*). The few eGFP+ SCPNs in medial cortex reside at the boundary between lateral and medial cortex. There is a significant difference in the number of tdTomato+ and eGFP+ SCPN across all three cortical locations, although the difference between the two groups in cingulate and medial cortex is more significant as compared with lateral cortex. Therefore, the majority of *Klhl14*^+^ SCPN reside in lateral cortex. These results also suggest that there may exist another subpopulation of lateral SCPN that are *Klhl14* negative. We also performed 3D AMaSiNe reconstructions of eGFP+ SCPN across three different Klhl14-Cre mice and find that all *Klhl14*+ SCPNs reside in lateral sensorimotor cortex (Fig. 2*J*, pink). Together, these results confirm that eGFP+ SCPNs are CSN_BC-lat_. This further indicates that Cre expression in Klhl14-Cre mice does reflect *Klhl14* expression and that the widespread recombination seen when using genomic Cre reporter mice likely reflects broader *Khl14* expression in embryonic neocortex.

Our recent results have found that there is additional diversity within CSN_BC-lat_ and that they comprise at least two additional subpopulations: (1) a subpopulation that only extends axons to the brainstem (CBN) and (2) a subpopulation of cervical-projecting CSN (CSNc), which reside interdigitated with CBN (Kaiser et al., 2025). We therefore investigated whether Klhl14-Cre is expressed by one or both these CSN_BC-lat_, i.e., whether Klhl14-Cre only labels a subset of CSN_BC-lat_. To test this, we first asked whether Klhl14-Cre+ SCPNs extend axons to the cervical spinal cord. If Klhl14-Cre is only expressed by CBN, conditional retrograde labeling from the cervical cord would not label any Klhl14-Cre SCPN. We therefore coinjected the identical combination of rAAVs as in Figure 2*A* from the cervical spinal cord at P2 (schematized in Fig. 2*B*). We injected rAAV to cover the entire extent of cervical dorsal funiculus that is spanned by the corticospinal tract to label all CSN. Since CSNs form a subset of SCPN, we find that overall, rAAV-CAG-tdTomato injection from the cervical cord labels fewer neurons than injections from the cerebral peduncle (Fig. 2 compare *A’*, *B’*). Importantly, however, we do find eGFP+ CSN labeled in Klhl14-Cre+ mice indicating that spinal-projecting neurons express Klhl14-Cre (Fig. 2*B’*,*B”*). 3D AMaSiNe reconstructions of eGFP-labeled CSN across three different Klhl14-Cre mice show that Klhl14-Cre CSN (Fig. 2*J*, blue neurons) occupy the same region in sensorimotor cortex as the overall population of Klhl14-Cre+ SCPN. Interestingly, these reconstructions show that Klhl14-Cre+ CSN occupy a smaller area in cortex as compared with Klhl14-Cre+ SCPN. This suggested that Klhl14-Cre is also expressed by a subpopulation of CSN_BC-lat_ that do not extend axons to the cervical spinal cord, i.e., CBN. To address this question, we also quantified the number of retrogradely labeled Klhl14-Cre+ SCPN versus Klhl14-Cre+ CSN (Fig. 2*K*,*L*). We find that there are significantly higher numbers of Klhl14-Cre+ neurons retrogradely labeled from the cerebral peduncle than the cervical spinal cord (Fig. 2*K*,*L*). This indicates that Klhl14-Cre is also expressed by neurons that extend axons to the cerebral peduncle but do not extend axons to the cervical spinal cord, i.e., CBN. Collectively, these quantitative results show that within the broad population of CSN_BC-lat_, Klhl14-Cre is expressed by both CBN and cervical-projecting CSN in lateral sensorimotor cortex.

Since our previous developmental *Klhl14* expression analyses were performed in the postnatal cortex (Sahni et al., 2021a,b), we could not accurately map areal locations of *Klhl14*+ SCPN within the cortex, since distinct cortical areas have not been fully defined at these early postnatal ages. Using injected Klhl14-Cre mice, we now had the ability to not only quantify the number of labeled neurons, but also their relative distribution across different cortical areas in the P14 mouse cortex. Consistent with our previous expression analyses that identified *Klhl14* is expressed in lateral sensorimotor cortex, we find that *Klhl14*+ SCPNs largely reside in somatosensory cortical areas (Fig. 2*K*,*L*). Together, these data reveal that Klhl14-Cre faithfully recapitulates our previously established *Klhl14* expression by CSN_BC-lat_ and that *Klh1l4*+ is expressed by both CBN and CSN in lateral sensorimotor cortex.

### Conditional anterograde labeling in Klhl14-T2A-Cre mice confirms that Klhl14+ CSN do not extend axons to the thoracic cord

Our prior work had confirmed that CSN_BC-lat_ do not extend axons beyond the cervical spinal cord to thoraco-lumbar levels (Sahni et al., 2021b). Given that *Klhl14* is expressed by CSN in lateral sensorimotor cortex, we next sought to investigate whether *Klhl14*+ CSN maintain the same axon targeting specificity in Klhl14-T2A-Cre mice. We therefore performed conditional anterograde labeling from lateral sensorimotor cortex. We coinjected at P0, the lateral cortex of Klhl14-Cre mice with two AAVs: (1) an AAV that constitutively expressed eGFP (AAV-hsyn-eGFP) and (2) an AAV that conditionally expressed tdTomato upon Cre-mediated recombination (AAV-FLEX-tdTomato). We first analyzed the brains from these injected mice at P15 and investigated expression of both reporters in cortex (strategy schematized in Fig. 3*A*). A series of coronal brain sections at four different rostrocaudal levels from one such injected mouse is shown in Figure 3*C*. We find that while the constitutive eGFP reporter expression spans much more broadly across lateral sensorimotor cortex, tdTomato expression remains confined to deeper layers. Even though the injections were performed in lateral cortex, in some mice, there is notable spread of eGFP (non-Cre-dependent reporter) expression into medial cortex (Fig. 3*D*,*D”*). However, in striking contrast, tdTomato (Cre-dependent reporter) expression does not spread into medial cortex (Fig. 3*E*,*E’*, dotted lines) indicating specificity of Klhl14-Cre expression to CSN_BC-lat_. 3D reconstruction of the cortical territories occupied by labeled neurons across multiple injected mice reveals substantial overlap between eGFP and tdTomato labeling across sensorimotor cortex; however, in some mice while eGFP+ neurons extend into medial cortex, tdTomato+ (Cre reporter) neurons remain confined to lateral cortex (Fig. 3*B*). We also quantified the spread of eGFP and tdTomato fluorescence in layer 5 at each of the four different rostrocaudal levels in three different injected mice (labeled “A,” “B,” and “C” in Fig. 3*F*; eGFP+ axons in these three mice displayed strikingly different extension in the spinal cord, which is discussed further in the next section). This quantitation revealed that although the areas of eGFP+ and tdTomato+ fluorescence were largely overlapping, eGFP fluorescence extended slightly more medially and laterally. For instance, eGFP fluorescence in Brains A and B spreads more medially at very specific levels (levels 1 and 2 in Brain A and level 3 in Brain B). eGFP fluorescence in Brain C does not spread medially beyond the area of tdTomato fluorescence at any of the four levels. In addition, eGFP fluorescence spreads more laterally in Brains B and C (levels 1 and 2 in Brain B and level 3 in Brain C). This further highlights the variability in spread of eGFP labeling, while tdTomato labeling remains consistent in lateral cortex across all rostrocaudal levels. The two AAVs used for the intracortical injections had identical titers as shown in Table 1. Therefore, these differences in spread cannot be explained by a difference in viral titers. Rather, these results highlight the specificity of Khl14-Cre expression by SCPN in lateral cortex.

We next analyzed axon extension in the spinal cord by comparing eGFP+ versus tdTomato+ CSN axons in axial spinal sections from P0-injected Klhl14-Cre mice at P15 (Fig. 4*A*). We find both eGFP+ and tdTomato+ axons are present in the cervical dorsal funiculus confirming that Klhl14-Cre is expressed by spinally projecting neurons in lateral sensorimotor cortex (Fig. 4*B–B”*). While some eGFP+ axons can be seen in the thoracic dorsal funiculus, likely originating from the medial cortex, i.e., CSN_med_ (as shown in Fig. 4*C”*), the majority of tdTomato+ axons are not present in the thoracic cord (Fig. 4*C*,*C’*). Ratiometric quantification of percentage of axons at T1–T2 over C1–C2 (T:C ratio) shows that there is more variability in the number of eGFP+ axons present in the thoracic dorsal funiculus than the number of tdTomato+ axons (Fig. 4*M*): 7 ± 3% of eGFP+ axons at C1 extend to T1, while only 1 ± 0.5% of tdTomato+ axons at C1 extend to T1. While this difference did not reach statistical significance (*p* = 0.0879 using the post hoc Mann–Whitney *U* test), these results clearly highlight that there is very little variation in the percentage of tdTomato+ axons that reach thoracic T1–T2 across 11 injected mice. Note the differences in percentage of eGFP+ axons at T1–T2 in the three mice analyzed in Figure 3*F* (highlighted as “A,” “B,” and “C” in Fig. 4*M*). While there were subtle differences in the cortical areas of eGFP versus tdTomato fluorescence, the number of labeled axons extending to the thoracic spinal cord varied markedly across the three mice (corresponding T:C % ratios of eGFP+ versus tdTomato+ axons for each of these mice were as follows: Brain A, eGFP+ axons: 20.4%, tdTomato+ axons: 2.9%; Brain B, eGFP+ axons: 7.6%, tdTomato+ axons: 0.4%; Brain C, eGFP+ axons: 0.3%, tdTomato+ axons: 0.2%). The higher T/C ratios of eGFP+ axons in the spinal cords of mice corresponding to Brains A and B are consistent with the slightly greater medial spread of eGFP fluorescence in the cortices of these two injected mice, since thoraco-lumbar-projecting CSN_TL_ reside in medial cortex (Sahni et al., 2021b). Collectively, these results highlight the relatively greater lack of specificity of labeling by only using AAV-eGFP injection into lateral cortex, which can still result in limited, but unintended transduction of thoraco-lumbar-projecting CSN_TL_. In contrast, labeling with Klhl14-Cre restricts transduction to cervical-projecting CSN.

We also analyzed three Klhl14-Cre mice that were injected at P0 with AAV-hsyn-eGFP + AAV-FLEX-tdTomato at P35 (schematized in Fig. 4*A*). Similar to the results obtained at P15, we find that there are significantly more eGFP+ axons in the dorsal funiculus at T1–T2 than tdTomato+ axons. Ratiometric quantification of percentage of eGFP+ versus tdTomato+ axons at T1–T2 in these mice were as follows: eGFP axons: 15 ± 3%; tdTomato axons: 5 ± 1% (*p* = 0.1 by Mann–Whitney *U* test). Interestingly, in these three mice the T:C % ratios of eGFP+ axons are all higher than the average ratio observed at P15, consistent with known evidence that there is greater AAV spread from the injection with time. Together, these quantitative results demonstrate that Klhl14-Cre+ CSN extend axons to the cervical spinal cord and not beyond, i.e., *Klhl14*+ CSNs are cervical-projecting CSN (CSNc) and that this early developmental specification persists into maturity. These results conclusively establish the specific expression of Cre by *Klhl14*+ CSN_BC-lat_ in Klhl14-Cre mice and highlight that this specificity enables selective targeting of CSN_BC-lat_ without solely relying on their spatial separation from CSN_med_ in the developing neocortex.

### Misexpression of Crim1 and Cbln1 in Klhl14-T2A-Cre mice redirects Klhl14+ CSN axons to thoracic cord

In our prior work, we identified two novel regulators of CSN axon extension—Crim1 (Sahni et al., 2021b) and Cbln1 (Song et al., 2023)—that when misexpressed in CSN_BC-lat_, can redirect these axons to extend past the cervical cord toward thoraco-lumbar segments. Since Klhl14-Cre provides a novel tool to selectively target CSN_BC-lat_, we next investigated whether Crim1 or Cbln1 misexpression can similarly redirect Klhl14-Cre+ CSNc axons. We coinjected the lateral cortex of Klhl14-Cre mice at P0 with two AAVs: (1) AAV-FLEX-tdTomato to label CSN_BC-lat_ axons and (2) either AAV-Crim1 or AAV-eGFP-2A-Cbln1. We could not use eGFP in AAV-Crim1 due to the limited packaging capacity of AAVs (Fig. 4*A*). We perfused these injected mice at P15 and analyzed the spinal cords from all three groups.

We analyzed axial spinal sections at cervical C1–C2 and thoracic T1–T2 comparing them with tdTomato+ (Klhl14-Cre+) CSNc axons in mice that received control AAV-GFP that limit their extension to the cervical cord (Fig. 4*B–C”*). In contrast, axial spinal sections from mice that were injected with either AAV-Crim1 or AAV-eGFP-2A-Cbln1 show substantial numbers of tdTomato+ (Klhl14-Cre+) axons extending to thoracic T1–T2 (Fig. 4*E–E”*,*G–G”*). Quantification of these results (Fig. 4*M*) confirmed that both *Crim1* and *Cbln1* can redirect *Klhl14*+ CSNc axons past their normal targets in the cervical cord toward the thoracic cord. We next analyzed the thoracic spinal cords from all three groups of injected mice. As expected, *Klhl14*+ CSNc axons in mice that received control AAV-GFP did not extend significantly into the thoracic cord (Fig. 4*J–J”*). In striking contrast, horizontal sections of thoracic cord spanning thoracic T2–T13, from mice that received either AAV-Crim1 or AAV-eGFP-2A-Cbln1, show *Klhl14*+ CSNc axons extending to far caudal levels of thoracic cord (Fig. 4*K–L”*). Together, these data indicate that known regulators of CSN axon extension that can redirect CSN_BC-lat_ axons are capable of similarly redirecting Klhl14-Cre+ CSNc axons. Further, Klhl14-Cre mice also offer a key advantage in these experiments. Our results confirm that Klhl14-Cre-labeled axons do not extend beyond the cervical cord (*n* = 11 mice as shown in Fig. 4*M*). Although we have limited statistical power due to the small sample number, these results suggest that a relatively small number of mice (*n* = 4 per overexpression group) can be used to detect significant effects of gene misexpression on CSN axon extension. These findings demonstrate that Klhl14-Cre mice will provide an efficient platform for screening additional genes affecting CSN_BC-lat_ axon extension using fewer animals.

### Klhl14-Cre+ SCPN exhibit identical projections in the brainstem as all CSN_BC-lat_

Our results with both anterograde and retrograde labeling show that Klhl14-Cre is expressed by CSN_BC-lat_ and that Klhl14-Cre+ SCPN span both CBN and CSNc in lateral cortex. Since retrograde labeling does not ensure that all neurons will be labeled from a given location, we also used anterograde labeling to investigate whether Klhl14-Cre preferentially drives Cre expression in either CBN or CSN in lateral sensorimotor cortex. We therefore compared axonal collateralization of Klhl14-Cre+ SCPN in the brainstem with those of anatomically labeled CSN_BC-lat_. Our hypothesis was that if Klhl14-Cre labels a subset of CSN_BC-lat_ in lateral sensorimotor cortex, then we would find specific brainstem regions that would be innervated by anatomically labeled CSN_BC-lat_, but not by Klhl14-Cre+ SCPN. We therefore analyzed brainstem sections from P15 Klhl14-Cre mice that had received coinjections of AAV-eGFP and AAV-FLEX-tdTomato into lateral cortex at P0 (Fig. 5*A*). We compared axonal collateralization by all CSN_BC-lat_ (eGFP+) with Klhl14-Cre+ SCPN (tdTomato+), spanning the entire rostrocaudal extent of the brainstem from midbrain to caudal medulla (Fig. 5*B*). Overall, we find very little difference between the two groups (Fig. 5*C–F*). In a few areas in the medial midbrain, we find very subtle differences in labeling intensity between the two groups of axons; however, overall, both CSN_BC-lat_ and Klhl14-Cre+ SCPN axons display very similar patterns of collateralization across the entire rostrocaudal axis. These findings indicate that Klhl14-Cre expression spans across the majority of SCPN in lateral sensorimotor cortex.

### An intersectional approach to target and manipulate Klhl14+ SCPN

Our results thus far have identified that Cre expression in P0 Klhl14-Cre mice delineates CSN_BC-lat_ in lateral sensorimotor cortex, even though there is broader Cre expression in embryonic neocortex. We therefore wanted to investigate whether this expression specificity at P0 could be used to specifically target CSN_BC-lat_ at maturity. Our approach of using conditional retrograde labeling using rAAVs specifically labeled Klhl14+ SCPN in lateral sensorimotor cortex, i.e., CSN_BC-lat_; however, for circuit-level functional investigations of Klhl14+ SCPN, e.g., via silencing or activation experiments using AAV-DREADDs, it would be imperative that this method of labeling CSN_BC-lat_ does not also target other *Klhl14*+ cells in the nervous system. We therefore checked whether our conditional retrograde AAV labeling from the cerebral peduncle (as shown in Fig. 2*A*) resulted in Cre-labeled neurons outside the cerebral cortex (schematized in Fig. 6*A*). As shown previously, this strategy labels eGFP+ (Klhl14-Cre+) SCPN in lateral layer V (Fig. 6*B*). We next analyzed coronal brainstem sections in these injected mice, i.e., near the rAAV injection site, to see if we could find any eGFP+ neurons. We find widespread labeling of cell bodies throughout the rostrocaudal extent of the brainstem (Fig. 6*C–D”*). These results indicate that while this approach of using conditional rAAV injections from the cerebral peduncles specifically labeled CSN_BC-lat_ in cortex, it also labels Klhl14-Cre+ neurons in the brainstem and therefore cannot be used to only target CSN_BC-lat_ in the CNS. Therefore, conditional Cre-dependent retrograde labeling from the cerebral peduncle at P0 in Klhl14-Cre mice cannot be used for circuit- and behavioral-level investigations of CSN_BC-lat_ function. To circumvent this problem, we next adopted a dual, intersectional viral labeling strategy to specifically label CSN_BC-lat_ without infecting Klhl14*-*Cre+ neurons in the brainstem.

We injected a rAAV-Cre-On-Flp-On-eYFP (ConFon-eYFP; Fenno et al., 2014) into the cerebral peduncles at P2, followed by an anterograde AAV-Flpo-mCherry injection into lateral cortex (strategy schematized in Fig. 6*E*). In this intersectional viral labeling approach, eYFP expression occurs only in neurons that express both Cre and FlpO. We next analyzed the cortex (Fig. 6*F*) and brainstem (Fig. 6*M*) from P15 Klhl14-Cre mice that had received these intersectional viral injections for eYFP labeled neurons. As expected, even though mCherry is expressed more broadly in the neocortex (Fig. 6*F*,*G*,*G”*,*I*,*I”*), we find that eYFP+ are restricted to layer V (Fig. 6*F*,*G’*,*I’*). Further, we find that eYFP+ neurons are present in lateral (Fig. 6*G’*,*I’*,*K’*) and not medial (*H’*,*J’*,*L’*) sensorimotor cortex. However, while Klhl14+ CSN_BC-lat_ axons are clearly both eYFP+ and mCherry+ in the pyramidal tract at the level of the ventral medulla (Fig. 6*M*,*N*,*N’*,*N”*), we find no eYFP+ cell bodies in the brainstem (Fig. 6*N’*,*O’*,*P’*). We similarly do detect CSN_BC-lat_ axon collaterals that are eYFP+ and mCherry+ in the spinal trigeminal nucleus (Fig. 6*P*,*P’*,*P”*), but no neuronal labeling in this region (Fig. 6 compare *P’*, *D”*). Therefore, this intersectional labeling approach enabled selective targeting of only CSN_BC-lat_ in the CNS without labeling Klhl14-Cre+ neurons in the brainstem. Our results show that Klhl14-Cre mice are a novel tool for the prospective identification and investigation of CSN_BC-lat_ axon targeting during development. In addition, intersectional viral labeling can expand their utility for functional analyses of CSN_BC-lat_ in the mature CNS.

## Discussion

The CST is a principal circuit for skilled voluntary motor control, which necessitates that SCPN axons make segmentally specific connectivity with their subcerebral targets. Our previous work had identified molecular delineation between developing SCPN subpopulations in sensorimotor cortex that will eventually extend axons to different levels of the neuraxis. *Crim1* expression could prospectively identify CSN_TL_, which suggested that this principle could be applicable to other subpopulations. In the present study, we establish that *Klhl14* expression, at specific developmental times, can prospectively delineate another CSN subpopulation—CSN_BC-lat_. These new results establish that developmental molecular delineation establishes a first step toward durable specificity of corticospinal connectivity at maturity. We further establish that Khl14-Cre provides a novel tool to reproducibly and reliably investigate CSN_BC-lat_ axon targeting with greater precision and specificity. This presents a significant advance over previous approaches that were reliant on anatomical separation of this subpopulation within sensorimotor cortex. Finally, we establish that, using intersectional viral labeling, Khl14-Cre can be used as a novel tool to specifically target CSN_BC-lat_ in the adult CNS. Our results indicate that molecular delineation early in development can be used to investigate corticospinal subpopulation-specific development, axon extension, connectivity, and eventually, function at maturity. Previous work identified that Klhl14 is expressed by spinal interneurons. We find that Klhl14-Cre is also expressed by neurons in the brainstem. Despite this widespread expression, our results show that using specific viral labeling approaches, both singly and in combination via intersectional viral labeling, Klhl14-T2A-Cre mice can still be used to investigate CSN_BC-lat_ in cortex with specificity. Future investigations will elucidate the applicability of this mouse line for investigating spinal interneurons, potentially using similar approaches. It is also intriguing to consider that there might be some shared anatomical characteristics of projections from neocortical and brainstem neurons and that is driven via the commonality of Klhl14 expression.

What makes Klhl14 a highly desirable tool to investigate SCPN subpopulation development and connectivity is that it is expressed with high specificity during development and is not expressed in adulthood. Several genes that exhibit highly specific expression during development are either absent in the mature cortex or even worse, become expressed more broadly in the adult brain. As a result, in such instances, adult gene expression cannot be used for targeting specific neuronal populations in the adult brain. Therefore, using developmental gene expression provides a valuable tool to target cell populations of interest, provided they can be accessed at the appropriate time in development. While inducible Cre lines do provide the ability for such temporal control, our approach shows that developmental anatomical labeling in Cre mouse lines can provide exquisite specificity for investigation and potential manipulation of SCPN subpopulations. Given the highly dynamic nature of gene expression during development, this is likely applicable to other neuronal populations beyond SCPN, indicating that genomic Cre reporters alone may be insufficient for both, investigating such parcellation and using it for functional manipulation.

Cre expression in Klhl14-Cre mice recapitulated our previously established specificity of *Klhl14* expression by CSN_BC-lat_; however, this specificity occurs at a specific developmental time. Interestingly, our results using genomic reporters find much broader Cre-dependent recombination indicating widespread Cre expression early in development, suggesting a potentially earlier role for Klhl14 in neocortical development. While it remains theoretically possible that this earlier Cre expression is driven by regulatory elements that are not associated with the Klhl14 locus, the specificity of expression at P0 suggests that this is likely not the case. Regardless, the approach of conditional labeling using AAV-mediated gene delivery enabled investigation of CSN_BC-lat_ with specificity. This further emphasizes that Klhl14-Cre expression in the embryonic CNS occurs too broadly, which in turn prevents its use with any genomic Cre reporters for any subsequent investigations/analyses that require specificity of expression. This also suggests that a similar principle could apply to other Cre lines that, when crossed to genomic reporters of Cre-dependent recombination, display broader expression that could be wrongly construed as nonspecific. A vast repertoire of Cre lines have now become available to the community (Gerfen et al., 2013; Daigle et al., 2018) and while there remain legitimate concerns regarding the specificity of expression (Stuber et al., 2015; Luo et al., 2020) and/or leaky transgene expression when using Cre-dependent labeling via AAVs (Botterill et al., 2021), our results indicate that discrepancy between results from such reporters and “established” expression data could also arise from spatiotemporal dynamics of gene expression. To conclusively establish gene expression requires integration across multiple approaches that can both qualitatively and quantitatively determine where and at what point in development a specific gene is expressed. Molecular strategies that integrate this critical information regarding gene expression dynamics across spatiotemporal dimensions are likely to be more precise and tailored for gene targeting and functional manipulation experiments.

Toward this goal, large-scale sequencing datasets are now becoming rapidly available, providing a valuable resource for using gene expression dynamics to gain molecular access to defined neuronal populations (Tasic et al., 2016; Rosenberg et al., 2018; Tasic, 2018; Di Bella et al., 2021; Yao et al., 2021). These datasets are beginning to provide greater resolution of such dynamics across the developing and adult CNS and presumably will provide greater insights into the necessary subtleties of gene expression that can be utilized to target specific cell types with precision. In line with this, in more recent experiments, we used single-cell transcriptomics (scRNA-seq) to investigate additional diversity within CSN_BC-lat_. Our results are that CSN_BC-lat_ comprises additional diversity, with both cortico-brainstem and cervical-projecting corticospinal neurons residing interdigitated in lateral sensorimotor cortex (Kaiser et al., 2025). ScRNA-seq finds that while *Klhl14* is expressed by both cortico-brainstem and corticospinal subsets, cervical-projecting corticospinal neurons express Klhl14 at a lower level (Kaiser et al., 2025). Consistent with this, our results using Klhl14-Cre mice highlight that *Klhl14* is expressed by both populations in lateral sensorimotor cortex, with a subset of Klhl14+ SCPN extending axons to the cervical spinal cord. Since retrograde labeling always has the caveat that it is impossible to label all neurons from any given level of the neuraxis, we used anterograde labeling, which is more sensitive, to investigate the extent of coverage of CSN_BC-lat_ by Klhl14-Cre. Analysis of axonal collateralization by Klhl14+ SCPN axons across the entire rostrocaudal extent of the brainstem indicates that Klhl14-Cre broadly labels CSN_BC-lat_ with extensive innervation across the brainstem and cervical spinal cord. Klhl14-Cre does not differentiate between cortico-brainstem and corticospinal neurons in lateral sensorimotor cortex, which we recently identified to be molecularly distinct (Kaiser et al., 2025).

Our quantitative data reveal that Klhl14-Cre+ SCPN early in development largely comprise cortico-brainstem neurons and a smaller subset of spinal-projecting neurons. scRNA-seq results show that corticospinal neurons in lateral cortex express lower levels of *Klhl14*, and therefore, it is possible that this is why we detect fewer Klhl14-Cre+ corticospinal neurons. It is, however, interesting to note that Klhl14+ SCPN includes a population of cervical-projecting corticospinal neurons even at maturity. It is well established that SCPN axons exhibit extensive pruning throughout development, such that several SCPN eventually lose their spinal projections (Stanfield et al., 1982; Stanfield and O'Leary, 1985; Abe et al., 2024). Therefore, significant numbers of anatomically labeled corticospinal neurons early in development can differentiate into cortico-brainstem neurons at maturity. Our results reveal that Klhl14-Cre+ corticospinal neurons maintain their spinal projection past this developmental period of axon pruning and therefore remain corticospinal neurons even at maturity.

*Klhl14* is only transiently expressed by SCPN during development, and single-cell sequencing datasets find that *Klhl14* expression is largely absent in adult cortex (Yao et al., 2021, 2023). Using retrograde labeling in Klhl14-Cre mice, we were therefore able to map the areal locations of Klhl14-Cre+ SCPN and CSN during development, at maturity. Consistent with the predictions from our previous developmental expression analyses, we find that the overwhelming majority of Klhl14-Cre+ SCPN and CSN reside in somatosensory cortex. While a small minority of Klhl14+ SCPN are annotated as located in primary motor cortex, these neurons actually reside in the transition zone between agranular motor cortex and granular somatosensory cortex (Tennant et al., 2011).

Since *Klhl14* expression is absent in adulthood, our results cannot establish whether Klhl14-Cre+ SCPNs maintain molecular distinctions from other SCPN subpopulations at maturity. However, recent single nuclear profiling across all descending spinal-projecting neurons in the mouse adult brain finds that spinal-projecting neurons within primary somatosensory and motor cortex are comparatively similar (Winter et al., 2023). This suggests that while Klhl14-Cre expression during development primarily delineates corticospinal projections arising from primary somatosensory cortex (Fig. 2*D*), these developmental molecular differences are not maintained into maturity. These results highlight the fact that developmental molecular controls over such differentiation are only expressed at the specific developmental time and that these molecular differences are largely not maintained into maturity. As a result, it becomes difficult to assign molecular identity to these developmentally defined neuronal populations past this developmental period of gene expression. Performing longitudinal molecular profiling of Klhl14-Cre+ SCPN through development into adulthood might identify potentially more subtle differences that could differentiate them from other SCPN subpopulations in the adult sensorimotor cortex. Using tools like Klhl14-Cre, along with the viral labeling approaches used in this study, we can now harness such developmental delineation of gene expression to investigate these subpopulations through the development into maturity and clearly define their developmental trajectory at anatomical, molecular, and functional levels.

We also established that Klhl14-Cre mice enable specificity of targeting CSN_BC-lat_ for analyzing axon targeting in the spinal cord. Even in instances where the AAV injection spread beyond the injection site in lateral cortex, axon targeting analysis showed that Klhl14-Cre+ axons remained restricted to the cervical cord. This specificity also enabled investigating molecular control over CSN_BC-lat_ axon targeting in the spinal cord. Consistent with the fact that Klhl14-Cre expression labels all CSN_BC-lat_, we find that misexpression of either of previously identified molecular regulators, *Crim1* and *Cbln1*, can redirect *Klhl14*+ CSN_BC-lat_ axons in a similar manner. Since CSN_BC-lat_ reside in lateral sensorimotor cortex, the strategy in previous experiments was aimed at restricting misexpression in CSN_BC-lat_ by injecting AAVs into lateral, but not medial, sensorimotor cortex (Sahni et al., 2021a,b; Song et al., 2023). Since AAVs are known to spread from the injection site, AAV injected into lateral cortex could potentially spread to medial cortex. This is especially likely in the developing cortex, where the spatial separation between these regions is smaller than in the adult. As a result, specifically targeting AAVs to CSN_BC-lat_ using anatomical separation alone is experimentally challenging.

Consequently, in previous experiments, any mice in which AAV injections spread into medial cortex had to be excluded from analysis, because in these mice, the labeled axons were no longer exclusively CSN_BC-lat_. In contrast, Klhl14-T2A-Cre mice enable selective labeling of CSN_BC-lat_ making it possible to investigate CSN_BC-lat_ axon targeting even in instances where AAV injections spread into medial cortex. This therefore provides a significant advance over our previous strategy and will enable testing function of other genes in a relatively more high-throughput manner.

In addition, our previous work identified Klhl14 as likely functioning as a transcriptional repressor, whereby it represses expression of both Crim1 and Cbln1 in CSN_BC-lat_, which would otherwise direct these axons to caudal spinal segments (Sahni et al., 2021b; Song et al., 2023). Our results with Klhl14-Cre mice now conclusively establish that *Klhl14*+ SCPN axons are responsive to misexpression of either of these genes. These new results further validate and confirm these previously identified genetic interactions. This establishes that Klhl14 functions in CSN_BC-lat_ to represses CSN_TL_ gene expression and restrict axonal projections to proximal targets in the brainstem and spinal cord.

Our results lay the foundation for establishing and using novel Cre mouse lines for targeting and investigating specific CSN subpopulations through development into maturity. Such tools can then be used to analyze the molecular control over their development, their anatomical organization at maturity, and potentially specific, circuit-level functional roles in adulthood. The CST is a critical circuit that has been investigated for functional recovery in several instances of nervous system damage. Collectively, results from such CSN subpopulation-specific targeting and analyses can begin to elucidate mechanisms that not only control normal CST development but that can also be utilized to affect regeneration and plasticity in instances of disease and injury. Our results are beginning to lay the foundation for such investigations into links between CST development and adult CST repair.

## Synthesis

Reviewing Editor: Matthew Grubb, King's College London Decisions are customarily a result of the Reviewing Editor and the peer reviewers coming together and discussing their recommendations until a consensus is reached. When revisions are invited, a fact-based synthesis statement explaining their decision and outlining what is needed to prepare a revision will be listed below. The following reviewer(s) agreed to reveal their identity: Julie JEZEQUEL. Note: If this manuscript was transferred from JNeurosci and a decision was made to accept the manuscript without peer review, a brief statement to this effect will instead be what is listed below.

Both Reviewers are generally supportive of the manuscript, while also presenting several issues that need to be addressed for acceptance.

After consultation, we agreed that the following course of action is most appropriate:

Please respond to *all* of Reviewer's comments.

Please respond to as many of Reviewer 1's comments as possible. Especially ensure that you respond to their specific comments related to the support from presented data for stated conclusions. In particular:

- define objective criteria for medial vs lateral cortex, and provide quantitative data showing the distributions of labelled cells in each region (Figs 2,3,6). In Fig6 this can combine with the co-label analysis requested by R2 to make a quantitative description that fully supports the 'selective targeting' of the title.

- provide better images of rAAV injection sites to demonstrate specificity

- provide statistical analysis of the GFP vs tdT data in Fig3 and the data in Fig5B (see also R2's comment on this)

- clearly define 'maturity' or provide data at later timepoints

Reviewer 1:

Lustig et al., have generated a Klhl14-T2A-Cre knock-in mouse line based on previous evidence that Klhl14 is specifically expressed in CSN BC-lat neurons (Shani et al., 2021) compared to CSN med at P1. They crossed the Klhl14-T2A-Cre line with the Emx1-FlpO:Ai65 to characterize axonal projections of recombined Klhl14 cells, and found broad Cre expression in the brain that did not recapitulate CSN BC-lat distribution. The authors have then performed a series of experiments using a combination of anterograde, retrograde and intersectional viral strategies to demonstrate the potential of their mouse line to specifically target the CSN BC-lat subpopulation. They eventually show that they could target CSN BC-lat neurons not purely relying on anatomical segregation (injections in lateral cortex only) but by using an intersectional strategy.

I acknowledge the work the authors have put into this manuscript, however some important experiments and analysis are lacking to provide robust evidence that this mouse line can be used efficiently. Despite the fact that specific targeting of the CSN BC-lat subpopulation can only be achieved under very restricted conditions (P0-P1 time window and intersectional strategies only), I also have some main concerns about the characterization of the Klhl14-T2A-Cre mouse line itself. The authors need to make a bigger effort to thoroughly and objectively characterize their tool for it to be useful to the community.

Figure 1

Major points:

- The authors observed unspecific recombination when crossing the Klhl14-T2A-Cre line with the Emx1-IRES-FlpO;Ai65. Did they further investigate what cell types were tdTomato+? What is the fraction of L5 pyramidal neurons labelled? Are only pyramidal cells labelled or other cell types as well? The authors rely purely on descriptive information here.

- The authors justify the use of this triple transgenic strategy since "Klhl14 is expressed by a subpopulation of spinal interneurons". The rationale behind this choice is sensible, however it is hard to apprehend the reliability of the Klhl14-T2A-Cre mouse line without a basic and rigorous characterization of their tool. The first step would be to simply cross their mouse line with a Cre-dependent reporter line and compare these data with the current ones (Emx1-IRES-FlpO;Ai65). Such analysis would provide precious information regarding their tool as it would 1) give the full distribution of Klhl14 expressing cells, 2) estimate the fraction of spinal interneurons expressing the gene of interest and the distribution of their axonal projections across the different spinal segments and 3) determine how much overlap there is between CSN BC-lat projections and spinal interneurons. This information is currently lacking in the manuscript.

- The authors propose that the lack of specific recombination in the Klhl14-T2A-Cre mouse line (broad recombination instead of specific to L5 CSN BC-lat pyramidal neurons in the lateral part of the cortex) is due to 1) a broad expression of Klhl14 in the embryonic cortex or 2) other genomic regulatory elements driving Klhl14-Cre expression.

Why did the authors not better characterize Klhl14 expression across pre- and postnatal development, which would directly tackle this question? An in-situ hybridization image of Klhl14 RNA expression at P4 is included as Figure 1C. However, this data originates from a previous study (Sahni et al., 2021) performed under different experimental conditions. A thorough characterization of Klhl14 expression over time would allow to determining whether its expression is restricted to CSNBC-lat or also present in other cell types before P0, as well as assess whether it is expressed in non CSN BC-lat cells beyond P1.

- FlpO is known to be a less reliable recombinase than Cre. Have the authors controlled that the heterozygous background of the experimental mice used here did not affect the level and efficiency of the Ai65 recombination? Their previous work used the Emx1-IRES-FlpO;Ai65 reporter line in an homozygous background (Sahni et al., 2021).

Minor points:

- Can the authors comment why they chose not to develop an inducible line, knowing that Klhl14 expression was dropping very quickly after P1 and/or was potentially expressed in other cells than CSN BC-lat neurons?

- Can the authors precise in the text which brains areas express tdTomato+ cells? They only mention that they cover all cortical layers and spread across the entire medio-lateral axis. Do some subcortical areas also express tdTomato+ cells?

Figure 2

Major points:

- How do the authors segregate medial from lateral cortex? The anatomical boundaries they used are not mentioned in the manuscript nor clearly appear on the figures.

- The authors conclude that eGFP+ SCPN (labelled by a rAAV-FLEX-eGFP) are restricted to the lateral sensorimotor cortex. Images from panels A' and B' show the presence of GFP+ cells in the medial part of the cortex. What is the proportion/density of GFP+ cells in lateral vs medial cortex? The authors should provide quantitative data to support their conclusion, which is purely qualitative and based on observations from low magnification images.

- How do they control for variability in viral infections? The number of cells infected via rAAV can vary a lot depending on multiple parameters such as the precision of the injection (targeting the right and enough axons), the virus efficiency/titer, the promoter driving the expression, etc.

- On the same line, how restricted to a specific spinal segment rAAVs injections are? Is there any possibility that peduncle injections spread to lower segments? Whole brain images give an indication but are hard to read. It would be very helpful to provide representative images of the injection sites with higher magnification to ensure that there is no spreading across several spinal cord areas.

Minor points:

- For more clarity, it would help in panel B to write "cervical spinal retrograde" instead of "spinal retrograde".

- The low magnification images in panels A' and B' do not allow to properly see the distribution of infected cells.

- Information about the rAAV-eGFP and rAAV-tdTomato are missing in the manuscript, only details about AAVs are provided.

Figure 3

Major points:

- The authors wrote, "tdTomato expression remains confined to the lateral cortex." Again, low magnification images are quite hard to read and do not allow to confidently validate this statement. For instance, Figure 3D' shows a decent amount of tdTomato+ cells in L6 of medial cortex (and a few in upper layers). As previously mentioned, clearly defining boundaries between medial and lateral cortex would help. More importantly, quantitative analysis showing the density of tdTomato+ and eGFP+ cells in lateral versus medial cortex should support the authors conclusions.

- The authors state "there is significantly more variability in the number of eGFP+ axons present in the thoracic dorsal funiculus than the number of tdTomato+ axons", and then conclude that this is due to "the greater lack of specificity of labelling AAV-eGFP in lateral cortex". First, no statistical analysis supports this conclusion. Furthermore, only 3 GFP mice out of 9 seem to have higher T:C ratios, the other 6 show comparable ratios to the tdTomato condition. How do the authors justify this? Could these 3 mice have more medial injections, hence more axons spreading to the thoracic segment?

- Have the authors tried to inject the AAV-FLEX-tdTomato in the medial cortex and look whether 1) there is any recombined cells present, and 2) tdTomato+ axonal projections can be found in the thoracic segment? The absence of these 2 would provide strong evidence that the Klhl14-T2A-Cre mouse line indeed allows to specifically target CSN BC-lat neurons.

Minor points:

- Are AAV-eGFP and AAV-FLEX-tdT injected simultaneously? This information is not clearly indicated in the methods. If they are injected separately, how can the others ensure comparable injection sites?

Figure 5

Major points:

- Why are there so many mice in the control condition compared to Crim1 and Cbln1 OE? How were the experiments performed? Are these animals littermates or have been performed in independent experiments? Once again, a statistical analysis is missing to support the authors' conclusion.

Figure 6

Major points:

- The authors mention that they "wanted to investigate whether this expression specificity at P0 could be used to specifically target CSNBC-lat at maturity". The title of the manuscript also refers to this however no experiment goes beyond P15. Can the authors further explain what they mean by "through development into maturity"?

- What are these Klhl14+ cells in the brainstem? Where do they send projections to? Does it matter if they do not overlap with projections from CSNBC-lat?

- The authors here used an intersectional strategy to prevent recombination from Klhl14+ cells in the brainstem. However, the brainstem is not the only source of Klhl14+ cells as previously demonstrated in this manuscript. If there are so many other cell types expressing Klhl14, to which extend this mouse line can be used?

Reviewer 2:

The authors generated Klhl14-T2A-Cre knock-in mice and used these mice with viral labeling in a specific developmental window as a new approach to investigate CSN(BC-lat) neuron. They managed to redirect Klhl14+ CSN axons to thoracic cord by expressing molecular regulators Crim1 and Cbln1 in these Klhl14-T2A-Cre mice.

I have the following major comments:

1. The images in figure 3 E-F are reused in figure 5C, and the data in figure 3G tdTomato group is reused in figure 5B control group. I suggest combining figure 3 and figure 5. The repeated images should be removed. And figure 3G and figure 5B could be combined into one bar graph and the eGFP signals in the Cbln1 group should also be included. The corresponding part in the manuscript should also be edited to avoid confusion.

2. Statistics needs to be done for the combined bar graph mentioned in 1. Please list the statistic results even if there are no significant differences.

3. What is the proportion of Klhl14+ neurons that project to Medulla? How specific is the conditional retrograde labeling of Klhl14+ neurons? Please show analysis of the colocalization of YFP and mCherry in figure 6 in both the cortex and the medulla.

4. Please use color blind friendly color palette in both microscopy images and schematics, avoid using green and red combination. Please change the color of figure 1A, 1B, 2A-A", 2B-B", 3, 4A and 5A.
